# Evolutionary remodeling of a remnant GET pathway factor into PEX38, an essential peroxin

**DOI:** 10.1073/pnas.2533726123

**Published:** 2026-02-26

**Authors:** Chethan K. Krishna, Stefan Gaussmann, Hirak Das, Martin Jung, Silke Oeljeklaus, Michael Sattler, Bettina Warscheid, Vishal C. Kalel, Ralf Erdmann

**Affiliations:** ^a^Department of Systems Biochemistry, Institute of Biochemistry and Pathobiochemistry, Faculty of Medicine, Ruhr University Bochum, Bochum 44780, Germany; ^b^Institute of Structural Biology, Molecular Targets and Therapeutics Center, Helmholtz Munich, Neuherberg 85764, Germany; ^c^Bavarian Nuclear Magnetic Resonance Center and Department of Bioscience, Technical University Munich School of Natural Sciences, Technical University of Munich, Garching 85748, Germany; ^d^Biochemistry II, Theodor Boveri-Institute, Biocenter Faculty of Chemistry and Pharmacy, University of Würzburg, Würzburg 97074, Germany; ^e^Department of Medical Biochemistry and Molecular Biology, Saarland University, Homburg 66421, Germany

**Keywords:** peroxisomes, glycosomes, PEX19, PEX38, peroxisomal membrane proteins, guided entry of tail-anchored proteins (GET)

## Abstract

PEX19 is a cytosolic receptor that directs membrane proteins posttranslationally to peroxisomes, as well as to mitochondria, lipid droplets, and the endoplasmic reticulum. A comprehensive *Trypanosoma* PEX19 interactome analysis uncovered PEX38 as an essential Euglenozoa-specific peroxin. PEX38 contains distinct domains that bind the cochaperone Hip and the PEX3-binding motif of PEX19, suggesting a role in stabilizing membrane proteins and preventing premature membrane docking. PEX38 illustrates functional repurposing in organelle biogenesis. It originated from a remnant of the GET/TRC pathway, typically responsible for the targeting of tail-anchored (TA) proteins to the endoplasmic reticulum. While most components of this machinery are absent in Euglenozoa, PEX38 has been retained and adapted to mediate peroxisomal membrane protein targeting. This evolutionary adaptation is unique to Euglenozoa. Because the PEX19–PEX38 interaction is essential for parasite viability and PEX38 has no human homologs, this complex is a promising therapeutic target against trypanosomatid parasites.

## Significance

Neglected tropical diseases affect over 1.5 billion people worldwide, with trypanosomatid parasites responsible for three major diseases: African sleeping sickness, Chagas disease, and leishmaniasis. These parasites rely on glycosomes, specialized peroxisomes essential for survival, making glycosome biogenesis an Achilles’ heel for therapeutic intervention. PEX38, an essential parasite-specific peroxin absent in humans, is the first peroxin discovered outside yeast or mammals and plays a unique role in protein targeting to glycosomes, representing a promising therapeutic candidate. The research provides insights into the mechanisms of membrane protein folding and their targeting during organelle formation. These findings highlight the potential for therapeutic strategies that exploit evolutionary differences between parasites and humans.

Approximately 30% of the proteome comprises of membrane proteins, which must be accurately sorted to their target membranes and inserted with the correct topology. This requires elaborate machineries, which are more complex in eukaryotes with membrane-bound subcellular organization. Membrane proteins are targeted to their destination membranes either cotranslationally or posttranslationally. Cotranslational transport prevents the release of hydrophobic membrane proteins into the cytosol, where they could aggregate or become toxic. However, certain membrane proteins, such as tail-anchored (TA) proteins, require posttranslational targeting. To prevent mistargeting or aggregation, some membrane proteins are translated in close proximity to their target organelle, a process known as organelle-associated translation (1).

PEX19 acts as a cytosolic receptor for newly synthesized peroxisomal membrane proteins (PMPs), which are recognized through their membrane peroxisome targeting signal (mPTS). Its flexible N-terminal region contains a PEX3 binding motif that is essential for docking at the peroxisomal membrane, whereas the structured C-terminus mediates binding to the PMPs (2). PEX19-bound PMPs are targeted to the peroxisomal membrane through binding to the docking factors PEX3 and PEX16 (3, 4). Subsequently, the PMP cargo is inserted into the membrane, and PEX19 is released to engage in a new import cycle. Despite this general model, the exact mechanisms governing PMP insertion and receptor release remain poorly understood.

PEX19 has been proposed to function not only as a receptor but also as a chaperone for its PMP cargo, based on two main observations, i) PMPs are unstable in the absence of PEX19, and ii) in vitro synthesized PMPs are stabilized in presence of recombinant PEX19 (5, 6). However, this chaperone-like role warrants reevaluation. Notably, PMPs also exhibit instability in the absence of PEX3 and/or PEX16, suggesting that PMP degradation may result from mislocalization to the cytosol in the absence of peroxisomal membranes, rather than from the absence of PEX19 per se (7). Furthermore, the reported stabilization of the in vitro synthesized PMPs in the presence of recombinant PEX19 could also include contributions from the unknown components of reticulocyte lysates used in the study. Moreover, PEX19 lacks sequence or structural similarity to canonical chaperones/HSPs, suggesting that PEX19 may not act as a chaperone in the classical sense. PEX19 client proteins contain single or several transmembrane domains (TMDs), with TMDs located near N or C termini or dispersed throughout the cargo proteins. A chaperone can function as holdase (ATP-independent, preventing accumulation of misfolded proteins), foldase (ATP-dependent, actively assist transition of misfolded proteins back to their native conformation), or disaggregase (8). It is likely that PEX19 functions only as holdase, preventing the aggregation of membrane proteins, as shown for huntingtin (9). Active folding of the membrane proteins has not been demonstrated for PEX19. For targeting PMPs with multiple TMDs, PEX19 is unlikely to function alone.

PEX19 primarily targets membrane proteins to peroxisomes, but it also facilitates the targeting of proteins to mitochondria, ER, and lipid droplets (5, 10). Mutations in PEX genes lead to peroxisome biogenesis disorders (PBDs), which result in severe neurological abnormalities and early death. These defects are exacerbated by damage to the mitochondrial integrity caused by mislocalization of PMPs to the mitochondria in the absence of peroxisomal targeting (7). Furthermore, pathogens, including viruses, can exploit the peroxisomal import machinery to enhance their survival, for example by directing viral proteins to peroxisomes via interaction with PEX19 (11, 12). Therefore, dissection of the early steps in PEX19–PMP targeting warrants investigation.

All trypanosomatid parasites possess single-membrane-bounded peroxisome-related organelles known as glycosomes (13, 14). This unique organelle compartmentalizes glycolytic enzymes and is involved in essential metabolic pathways, including gluconeogenesis, pentose phosphate pathway, purine salvage, pyrimidine biosynthesis, fatty acid elongation, and sterol biosynthesis (15). Given the critical role of glycosomes in trypanosomatid parasites, their biogenesis is an important therapeutic target.

In this study, we conducted a comprehensive analysis of the *Trypanosoma* PEX19 interactome by labelfree quantitative mass spectrometry. Among the top interactors identified were PEX38, a Euglenozoa-specific peroxin, and the Hsc70 interaction protein (Hip). Using peptide arrays, biochemical assays, and high-resolution NMR structural analysis, we demonstrate that PEX38 directly binds to the PEX3 binding motif of PEX19 and serves as a molecular bridge between PEX19 and Hip. A high-resolution NMR structure of the complex reveals molecular details for the interaction further supported by mutational analysis. These findings reveal a previously unrecognized mechanism by which PEX19 may access cytosolic chaperones for stabilizing membrane proteins and facilitating their glycosomal import. Interestingly, most canonical GET pathway components are absent in Euglenozoa. PEX38 appears to represent a repurposed remnant of this pathway, remodeled to function as a cochaperone that bridges the PEX19–PMP complex with the chaperone machinery. We show that both PEX38 and its interaction with PEX19 are essential for parasite viability. Notably, this interaction is absent in the human counterpart making PEX38 a promising candidate for structure-based drug development against trypanosomatid parasites.

## Results

### PEX19 Interactome Analysis Identifies PEX38 as Predominant Cytosolic Binding Partner.

**Fig. 1.**
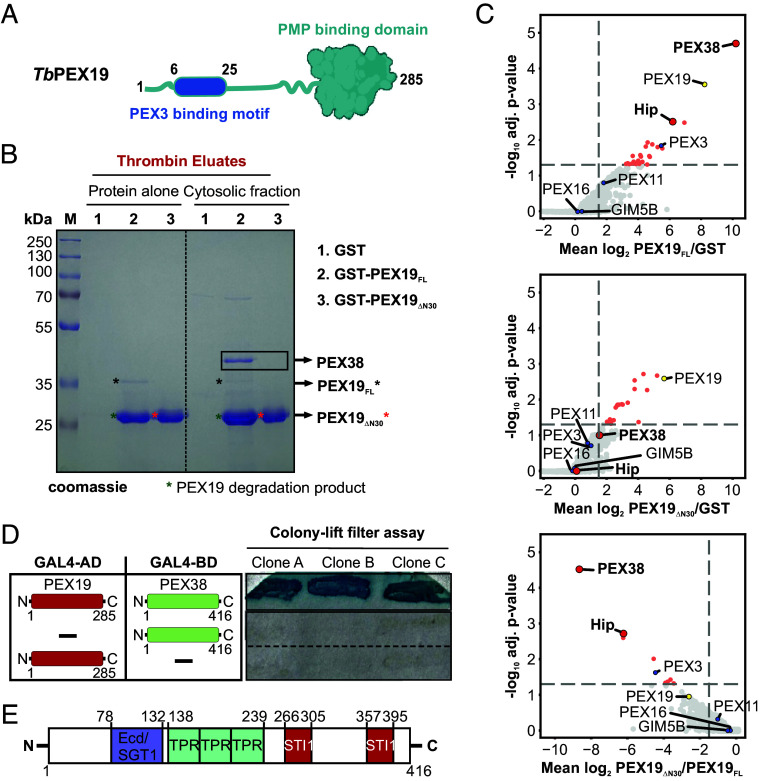
Identification of PEX38 as a dominant binding partner of PEX19. (*A*) Domain architecture of *Tb*PEX19 showing the C-terminal PMP binding domain (green) and N-terminal unstructured region with PEX3 binding motif (blue). (*B*) Affinity pulldown of *T. brucei* cytosolic proteins with GST-PEX19, analyzed by SDS-PAGE and Coomassie staining; PEX38 is highlighted (black box). (*C*) Cytosolic interactomes of GST-PEX19_FL_, GST-PEX19_ΔN30_, and GST control analyzed by Affinity Purification MS. Volcano plots show MS-based iBAQ ratios of PEX19_FL_/GST (*Top*), PEX19_ΔN30_/GST (*Middle*), and PEX19_ΔN30_/PEX19_FL_ (*Bottom*) along with adjusted *p*-values determined by the rank-sum method. Axes: fold change (log_2_) and adjusted p-value (-log_10_). Highlighted proteins: PEX19 bait (yellow), known glycosomal PMPs (blue), PEX38/Hip (red), significant hits (light red). Vertical dashed lines: log_2_ ratio >1.5 (*Top* and *Middle*) or <1.5 (*Bottom*), n ≥ 2 replicates; horizontal line: 5% false-discovery-rate (n = 3 replicates). (*D*) Y2H colony-lift filter assay confirms PEX19–PEX38 interaction (n = 3 biological replicates, 3 clones each). (*E*) Domain architecture of PEX38 showing ECD/SGT1, TPR, and STI1 domains.

To identify interaction partners of PEX19, we performed affinity pull-down assays using recombinant GST-tagged *T. brucei* PEX19 (full-length i.e., FL or lacking PEX3 binding region i.e., ΔN30; purification profile shown in *SI Appendix*, Fig. S1) in combination with cytosol or solubilized organellar fraction obtained from *Trypanosoma* parasites (Fig. 1*A* and *SI Appendix*, Fig. S2*A*). PEX19 and the bound proteins, eluted using thrombin cleavage, were analyzed by SDS-PAGE with Coomassie (Fig. 1*B*) and silver staining (*SI Appendix*, Fig. S2*B*), which revealed potential binding partners as additional bands in comparison to the GST control. Notably, a prominent cytosolic binding partner of ~38 kDa was detected in the Coomassie stained eluate of PEX19_FL_ but was almost absent in the eluate of PEX19_ΔN30_ (Fig. 1*B*). This protein was unlikely to be PEX3 itself, since it is a membrane protein of a predicted molecular size of 52 kDa.

The affinity-purified complexes were analyzed by label-free quantitative mass spectrometry (MS) to define the cytosolic and organellar PEX19 interactomes. From the 2,361 total identified protein groups, we calculated protein abundance ratios between PEX19 and the GST control (PEX19_FL_/GST), as well as the ratios between the two PEX19 variants (PEX19_ΔN30_/GST and PEX19_ΔN30_/ PEX19_FL_). Proteins significantly enriched with both PEX19 variants from cytosolic (Fig. 1*C*) and organellar fractions (*SI Appendix*, Fig. S2*C*) were determined based on rank-sum statistical analysis (n = 3, adj. p value < 0.05, mean ratio > 2.25). Of the 24 potential interaction partners of full-length PEX19 in the cytosol, a protein with TriTrypDB ID “Tb427_060045000” was the most significantly enriched (Fig. 1 *C*, *Top* panel; Dataset S1). Orthogonal validation by yeast two-hybrid (Y2H) analysis also revealed a clear interaction of this protein with full-length PEX19 (Fig. 1*D*). Based on its strong interaction with PEX19 and subsequent characterization, we designated this protein as PEX38. The two other highly enriched proteins for PEX19_FL_ in the cytosol were Hip and PEX3.

Similarly, among the 45 potential PEX19 interactors identified in the organellar fraction, PEX38 was the most significantly enriched protein after PEX3 (*SI Appendix*, Fig. S2 *C*, *Left* panel). Interestingly, this interaction was abolished in the truncated PEX19 variant lacking the N-terminal 30 amino acids (PEX19_ΔN30_) in both cytosol (Fig. 1*C*, *Middle* panel) and organellar fractions (*SI Appendix*, Fig. S2 *C*, *Middle* panel), suggesting that PEX19 requires its extreme N-terminal region for binding to PEX38. To further validate this observation, we compared the protein abundance between the two PEX19 variants and found that both PEX38 and Hip were depleted in the cytosolic fraction of PEX19_ΔN30_ relative to full-length PEX19 (PEX19_FL_) (Fig. 1 *C*, *Bottom* panel). Likewise, PEX38 and known PMPs like PEX3 and PEX16 were depleted in the organelle-enriched fraction (*SI Appendix*, Fig. S2 *C*, *Right* panel). The top binding partners of both variants in the cytosolic and organellar fractions are listed in Dataset S1.

Bioinformatic analysis of *Tb*PEX38 using BLAST revealed yeast Sgt2 and human SGTA/SGTB as its closest homologs. Both PEX38 and human or yeast SGT proteins share a tetratricopeptide (TPR)-like helical superfamily domain and belong to the SGT (Small glutamine-rich TPR repeat-containing) protein family (Fig. 1*E* and *SI Appendix*, Fig. S3*A*). However, *Trypanosoma* PEX38 protein displays notable differences compared to the human and yeast SGT proteins. The N-terminal “SGTA homodimerization domain” (InterPro ID: IPR032374, short name: SGTA dimer) conserved in SGT2/SGTA/SGTB is not detected in PEX38, and glutamine-rich region at the C-terminus, a characteristic name-giving feature of SGT proteins is also absent in PEX38. Instead, *Tb*PEX38 contains a region with similarity to the Ecd (Ecdysoneless) domain found in human Ecd (hECD), also known as SGT1 (Suppressor of GSR2 1) (Fig. 1*E*). *Tb*PEX38 also contains two STI1 (STress Inducible 1) domains, which represent heat shock chaperonin-binding regions, a feature not detected in human *Hs*SGTA/B and yeast *Sc*Sgt2 via InterPro scan. Overall, *Tb*PEX38 shares relatively low sequence identity (~24 to 31%) with *Hs*SGTA/B or *Sc*Sgt2 with the highest conservation localized to the TPR domain (*SI Appendix*, Fig. S3 *B* and *C*). Consistent with these structural or domain differences, Y2H analysis showed no detectable interaction between the *Hs*PEX19 and *Hs*SGTA, while a weak interaction was observed for *Sc*PEX19 and *Sc*Sgt2 (*SI Appendix*, Fig. S4*A*). Although *Tb*PEX19 shares relatively low sequence homology with host *Hs*PEX19, its PEX3-binding motif is conserved (16). Therefore, *Tb*PEX19 was also tested with host *Hs*SGTA and *Sc*Sgt2, but no interaction was observed (*SI Appendix*, Fig. S4*B*). These distinctive structural features and experimental results underscore the uniqueness of PEX38 protein to trypanosomatids.

### Characterization of the PEX19–PEX38 Protein–Protein Interaction (PPI).

**Fig. 2.**
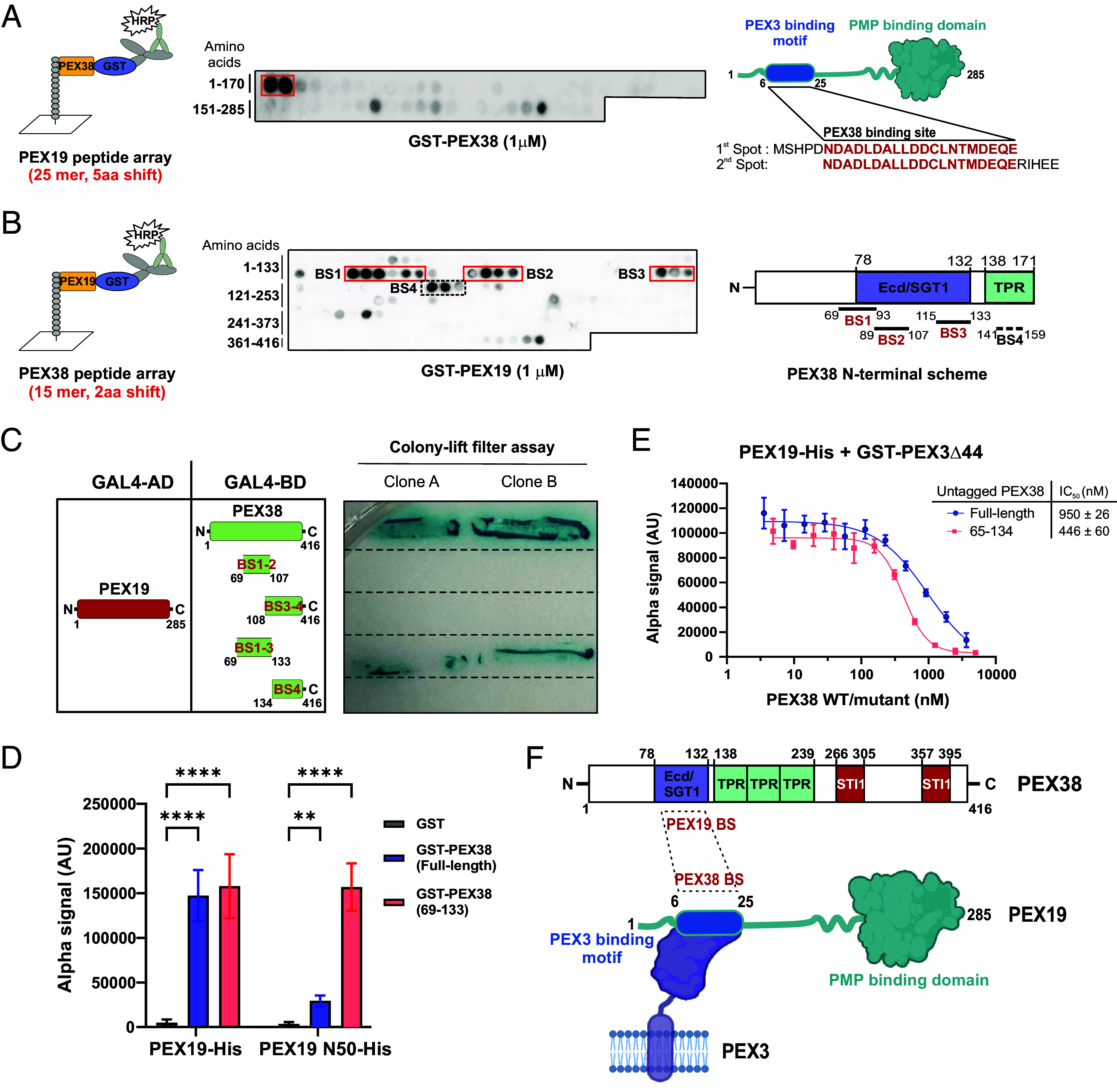
Cytosolic PEX38 and membrane docking protein PEX3 bind to the N-terminal helix of PEX19. (*A*) Peptide array scheme (*Left*) and chemiluminescent detection of GST-PEX38 binding to PEX19 peptides (*Middle*; binding site in red box; GST control, *SI Appendix*, Fig. S5*A*). Identified binding site overlaps the known PEX3 binding motif (*Right*). (*B*) PEX38 peptide array probed with GST-PEX19 (middle, binding sites in red/dotted boxes; GST control, *SI Appendix*, Fig. S5*B*). Four binding sites (BS-BS3, red; BS4, dotted) identified in the N-terminal domain of PEX38 (*Right*). (*C*) Y2H colony-lift filter assay validates PEX19 binding sites in PEX38 (n = 2 independent clones; controls, *SI Appendix*, Fig. S5*C*). (*D*) AlphaScreen binding assay for PEX19-His (full-length or 1 to 50) with GST-PEX38 (full-length or 69 to 133) or GST control. Statistical comparison by two-way ANOVA with the Šídák test: ****P < 0.0001; **P = 0.0012. Error bars, SD; n = 3 independent experiments, 3 technical replicates each. (*E*) AlphaScreen competition assay: untagged PEX38 (full-length, IC_50_ = 950 nM; 65 to 134, IC_50_ = 446 nM) displaces GST-PEX3Δ1-44 from PEX19-His. Mean ± SD, n = 3 independent experiments, 3 technical replicates each. (*F*) Schematic summary of PEX19–PEX38 interaction sites and known PEX19–PEX3 interaction.

Proteomic and Y2H analysis revealed a clear interaction between full-length *Tb*PEX19 and *Tb*PEX38 (Fig. 1). To further delineate the PEX19–PEX38 binding interface, we employed synthetic peptide arrays spanning the sequences of each protein. These arrays were probed with recombinantly purified GST-tagged versions of the interacting partner, followed by antibody detection of the probe-bound interaction peptide spots. On the *Tb*PEX19 array, two spots corresponding to the N-terminal region spanning amino acid residues 1 to 25 and 6 to 30 of PEX19 indicated potential binding by PEX38 (Fig. 2 *A*, *Middle* panel), in contrast to the GST control, which showed no binding in this region (*SI Appendix*, Fig. S5*A*). This N-terminal region (1 to 25 residues) of PEX19 is also known to mediate the interaction with PEX3 at the peroxisomal/glycosomal membrane, a critical docking step for targeting and membrane insertion of PMPs across different organisms (Fig. 2*A*, scheme on *Right*) (16). Similarly, using the PEX38 peptide array, we identified four potential PEX19 binding sites (BS1-BS4) in PEX38 (Fig. 2 *B*, *Middle* panel), compared to the control GST (*SI Appendix*, Fig. S5*B*). The identified PEX19 binding sites in PEX38 are located in regions comprising amino acid residues 69 to 93, 89 to 107, 115 to 133, and 141 to 159. Notably, the first three binding sites (BS1–BS3) are located within the Ecd/SGT1 domain, which is only present in PEX38 (*SI Appendix*, Fig. S3*A*), while the fourth binding region (BS4) is situated in the TPR domain (Fig. 2*B*, scheme on *Right*).

The PEX19 binding regions identified in PEX38 via peptide array analysis were further tested by Y2H analysis (Fig. 2*C*). The individually tested constructs, PEX38_69–107_ (BS1-BS2), PEX38_108–416_ (BS3-BS4), and PEX38_134–416_ (BS4), did not interact with PEX19 in Y2H. However, a construct PEX38_69–133_ comprising the conserved Ecd/SGT1 domain, which contains BS1-BS3, showed a clear interaction with PEX19, comparable to that observed with the full-length PEX38 (Fig. 2*C*), with negative controls confirming the absence of autoactivation (*SI Appendix*, Fig. S5*C*). BS4 could not be conclusively validated and may require additional experimental strategies to determine its contribution to the interaction.

As an independent approach, we assessed the PEX38–PEX19 interaction using a semiquantitative AlphaScreen-based assay (Fig. 2*D*). The assay revealed a strong interaction between full-length PEX19 (PEX19_FL)_ and both full-length PEX38 (PEX38_FL_) and the truncated PEX38_69–133_. A short N-terminal fragment of PEX19 (PEX19_1–50_) showed 10 times weaker interaction with PEX38_FL_. However, the same PEX19_1–50_ fragment displayed robust interaction with PEX38_69–133_, which bound PEX19_FL_ and PEX19_1–50_ with comparable affinity. In vitro pull-down experiments with the same proteins/fragments qualitatively confirmed these interactions (*SI Appendix*, Fig. S5*D*). Taken together, these data identify and validate PEX38–PEX19 PPI.

Docking of the PMP-bound PEX19 to the glycosomal membrane relies on the binding between PEX3 and the N-terminal region of PEX19. Since both PEX38 and PEX3 bind to the same region in PEX19 (Fig. 2 *A*, *Right* panel scheme), we investigated whether these two proteins compete for PEX19-binding. To this end, we performed an AlphaScreen-based displacement assay using equimolar concentrations of PEX19 and PEX3_Δ1–44_ proteins, while titrating increasing amounts of untagged PEX38 (FL or 65 to 134 constructs). Both PEX38 constructs were able to displace PEX3 from PEX19 in a dose-dependent manner (Fig. 2*E*). Quantitatively, ∼950 nM of PEX38_FL_ and ∼446 nM of PEX38_65–134_ were required to displace 30 nM of GST-PEX3Δ44, suggesting that PEX3 has a higher affinity for PEX19 than PEX38. This higher affinity is likely due to additional PEX3-binding regions outside of the PEX19 N terminus, as previously reported (16–18).

In conclusion: By combining cell fractionation with pull-down and quantitative MS analysis, we identified PEX38 as a cytosolic PEX19-binding protein that interacts with the N-terminal region (1 to 30) of PEX19, the same region involved in binding of PEX19 to the membrane docking factor PEX3.

### An Amphipathic Helix From PEX19 Interacts with a Helical Bundle of PEX38.

In previous peptide array experiments, we identified the N-terminal amino acid segments 1 to 25 and 6 to 30 within PEX19 to interact with a small domain of PEX38_65–134_ (Fig. 2*F*). To map this interaction and for high-resolution structural analysis, we generated a PEX19_1–50_ construct with a C-terminal SGGY extension to facilitate detection at 280 nm. We performed isothermal titration calorimetry (ITC) and NMR titrations to provide quantitative binding affinities and map the interaction at high resolution and finally determined the structure of the PEX38/PEX19 complex.

**Fig. 3.**
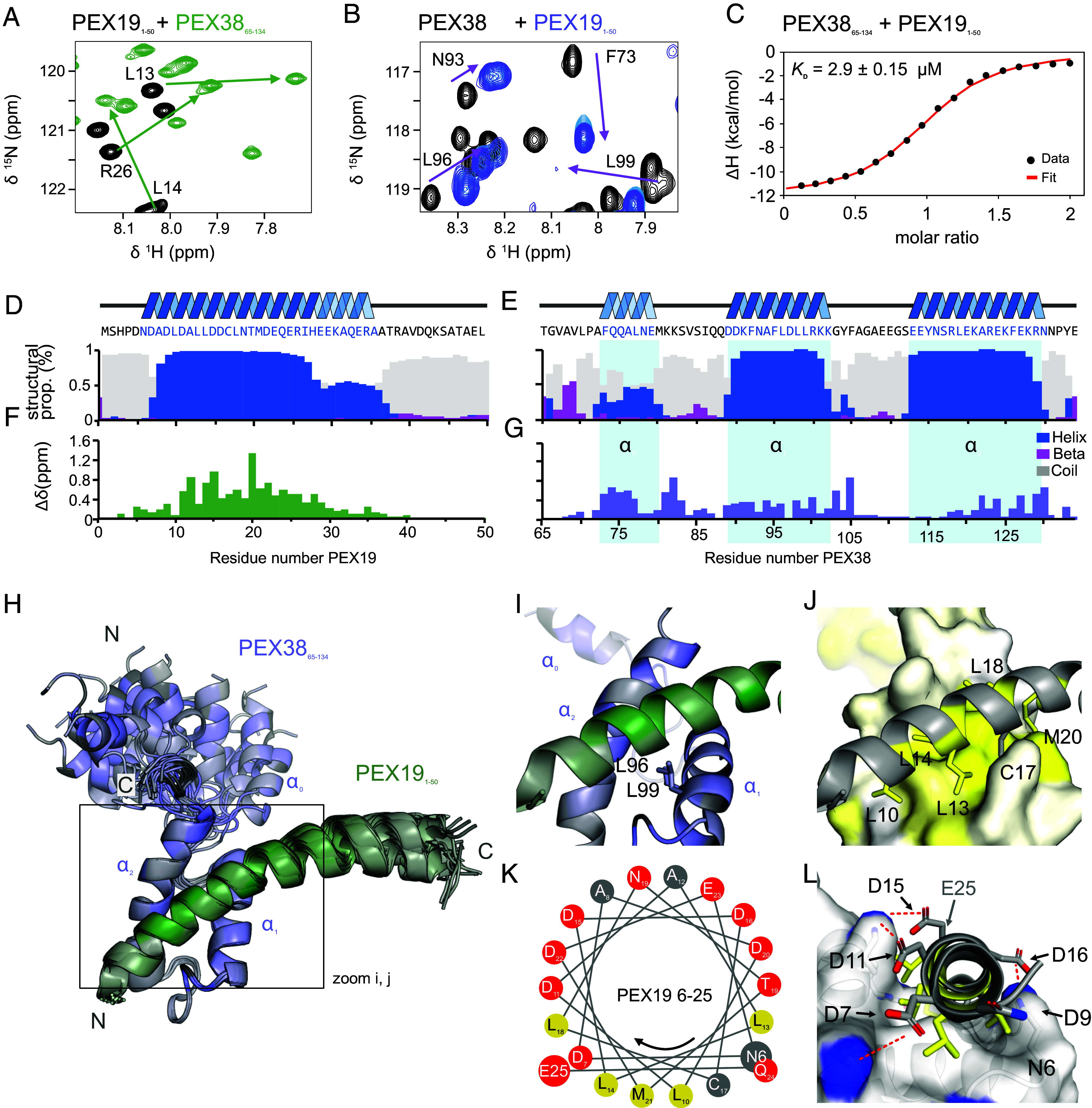
The induced amphipathic helix of PEX19_1–50_ binds to the hydrophobic core of PEX38_65–134_ helical bundle. (*A*) NMR titration of ^15^N labeled PEX19_1–50_ with unlabeled PEX38_65–134_ and (*B*) ^15^N labeled PEX38_65–134_ with unlabeled PEX19_1–50_ shows large chemical shift perturbations and line-broadening, indicating a strong interaction between the proteins (full spectra shown in *SI Appendix*, Fig. S6 *A*–*C*). (*C*) ITC experiments showing titration of PEX38 with PEX19. The experiment was conducted in technical triplicates. (*D* and *E*) Secondary structure propensities of PEX19_1–50_ and PEX38_65–134_ were predicted using TALOS-N (19), based on the secondary chemical shifts (*SI Appendix*, Fig. S6 *F* and *K*). The propensities for α-helix, β-sheet, and coil structures are shown in blue, magenta, and gray, respectively. The helical regions are also visualized above and mapped onto the PEX19 and PEX38 sequences. (*F* and *G*) Chemical shift perturbations (Δδ in ppm) of PEX19 saturated PEX38 or PEX38 saturated PEX19 plotted on the sequences of each protein. (*H*) Chemical shift perturbations (from panels *F* and *G*) mapped on an overlay of the 10 best-scored structures with trimmed flexible regions (PEX38_65–69_ and PEX19_39–50_). (*I*) A zoomed-in view of the first structure highlights the key positions of leucines L96 and L99, which are crucial for the interaction beween PEX38 and PEX19. (*J*) Surface representation of PEX38 with indicated hydrophobicity (20) displayed as white to yellow gradient. Residues from PEX19 L10, L13, L14, C17, L18, and M20 facing the hydrophobic surface of PEX38 are represented as colored sticks. (*K*) Schematic representation of the PEX19 amphipathic helix 6 to 25 flanked by N6 and E25. (*L*) A front view of the PEX19 amphipathic helix reveals charged residues D7, D11, D15, D16, and D22 (E25 is not visible here, please see *SI Appendix*, Fig. S7*C*) on the hydrophilic side, providing electrostatic interactions with positively charged lysine and arginine residues from PEX38. These contacts are represented by dashed red lines, and the amino groups of the PEX38 lysines are highlighted as blue surfaces. A complete representation is shown in *SI Appendix*, Fig. S7*C*.

NMR titration experiments were conducted using ^15^N-labeled PEX19_1–50_ titrated with unlabeled PEX38_65–134_, and reciprocally unlabeled PEX19_1–50_ into ^15^N-labeled PEX38_65–134_. Both setups revealed an intermediate to slow exchange binding regime, as evidenced by a decrease followed by an increase in peak intensities with increasing ligand concentration (Fig. 3 *A* and *B* and *SI Appendix*, Fig. S6 *A*–*C*). This behavior is consistent with the dissociation constant (*K*_D_) of approximately 3 µM determined by ITC (Fig. 3*C* and *SI Appendix*, Table S1). Notably, titration of unlabeled PEX19_1–140_ onto ^15^N-labeled PEX38_65–134_ induced similar effects to those observed in the PEX38 spectra, confirming that the binding site is indeed covered by PEX19_1–50_ (Fig. 3*A* and *SI Appendix*, Fig. S6 *A* and *B*). However, residue F73 exhibited significant line broadening during titration with PEX19_1–140_, suggesting a different role in binding to PEX19 full-length (FL) compared with PEX19_1–50_ (*SI Appendix*, Fig. S6*B*).

To gain insight into the secondary structure and confirm the binding interface at residue resolution, we recorded triple resonance experiments and assigned the backbone resonances of PEX38_65–134_ and PEX19_1–50_ in both their free and complex states, enabling assessment of secondary structure from secondary chemical shifts (Δδ^13^Cα-Δδ^13^Cβ). In the free state, PEX19_1–50_ is largely unstructured, with only regions of low helical propensity (*SI Appendix*, Fig. S6 *D* and *E*). Remarkably, when bound to PEX38_65–134_, PEX19_1–50_ adopts a stable helical structure in the region of amino acid residues 6 to 27 and a less populated helix from 27 to 37, while the remaining residues remain unstructured (Fig. 3*D* and *SI Appendix*, Fig. S6*F*). This binding-induced helix formation is further supported by strong chemical shift perturbations of the PEX19_1–50_ amide resonances when compared to free PEX19_1–50_ (Fig. 3 *A* and *F*).

The secondary structure analysis of free PEX38_65–134_ highlights three helical regions α_0_, α_1_ and α_2_ located at amino acid residues 72 to 79, 90 to 102, and 113 to 129, respectively. (*SI Appendix*, Fig. S6 *G* and *H*). The (α_0_) helix comprises of about two turns, and is about 50% populated, while the longer α_1_ and α_2_ helices, spanning 3 and 5 turns respectively, are fully populated (*SI Appendix*, Fig. S6 *G* and *H*). Upon binding PEX19_1–50_, the helical propensity of α_1_ and α_2_ increases slightly (Fig. 3 *E* and *G* and *SI Appendix*, Fig. S6 *J* and *K*), while the helical propensity of α_0_ decreases (*SI Appendix*, Fig. S6 *H* and *K*). To assess backbone flexibility on the pico- to nanosecond timescale, we recorded {^1^H}-^15^N heteronuclear NOE experiments, which showed increased rigidity in helices α_1_ and α_2_ but not in α_0_, upon binding of PEX19_1–50_ (*SI Appendix*, Fig. S6 *I* and *l*).

Next, we determined the NMR solution structure of the PEX38_65–134_-PEX19_1–50_ complex (*SI Appendix*, Fig. S7*A*), utilizing full side-chain assignments and multiple NOESY experiments. This provided 2,752 proton–proton distance restraints, including 224 intermolecular long-range restraints (*SI Appendix*, Fig. S7 *B* and *C* and Table S2). A comprehensive overview of the structure calculation statistics and quality assessment is provided in *SI Appendix*, Tables S2 and S3, respectively. Analysis of the calculated complex confirmed that the α_0_ region of PEX38_65–134_ and the disordered region of PEX19 (37 to 50) do not contribute to the complex structure, as shown by transparency or omission in subsequent figures (Fig. 3*H* and *SI Appendix*, Fig. S7*B*). The structures reveal two key leucine residues, L96 and L99, in the hydrophobic core of PEX38_65–134_, which exhibited significant chemical shift perturbations and line-broadening (Fig. 3 *A* and *I*). L96 mediates contacts with the second helix (α_1_) and a loop region, while L99 resides at the hydrophobic binding interface between PEX38 and PEX19, explaining the observed perturbations and line-broadening. The hydrophobic surface of PEX38 (Fig. 3*J*) is occupied by hydrophobic residues L10, L13, L14, C17, L18, and M20 from the amphipathic helix formed by PEX19_6–25_ (Fig. 3 *J* and *K*). On the hydrophilic face of the amphipathic helix, flanked by N6 and E25, multiple aspartates (D7, D11, D15, and D16) and one glutamate (E23) confer a negative charge, enabling additional salt bridge contacts with positively charged side chains of neighboring Lys residues (K102, K121, K125) and R129 on the PEX38 side (Fig. 3 *K* and *L* and *SI Appendix*, Fig. S7*C*).

Building on our structural insights, we sought to disrupt the PEX19–PEX38 interaction through targeted mutagenesis. Given the numerous intermolecular NOEs between the α_1_ helix of PEX38_65–134_ and PEX19_1–50_, we performed a “proline walk” mutagenesis on a synthetic 15-mer peptide spanning the α_1_ (98 to 102) (*SI Appendix*, Fig. S8*A*). Notably, substituting any of the central residues FLDLL (95 to 99) with proline completely abolished its interaction with PEX19 (*SI Appendix*, Fig. S8*B*). This was further confirmed by the Y2H assay, where we introduced proline substitutions to disrupt the helical structure (L96P or LDL95-98PPP), or aspartic acid substitution to disturb the hydrophobic core (L96D). Wild-type PEX38_FL_ as well as PEX38_69–133_ interact with PEX19, while all three mutations of PEX38 completely abolished this interaction (*SI Appendix*, Fig. S8 *C* and *D*). These results corroborate our structural data and underscore the critical role of the helical structure and the hydrophobic core for PEX19 binding (Fig. 3 *I* and *J*).

### PEX38 Is a Predominantly Cytosolic Protein.

**Fig. 4.**
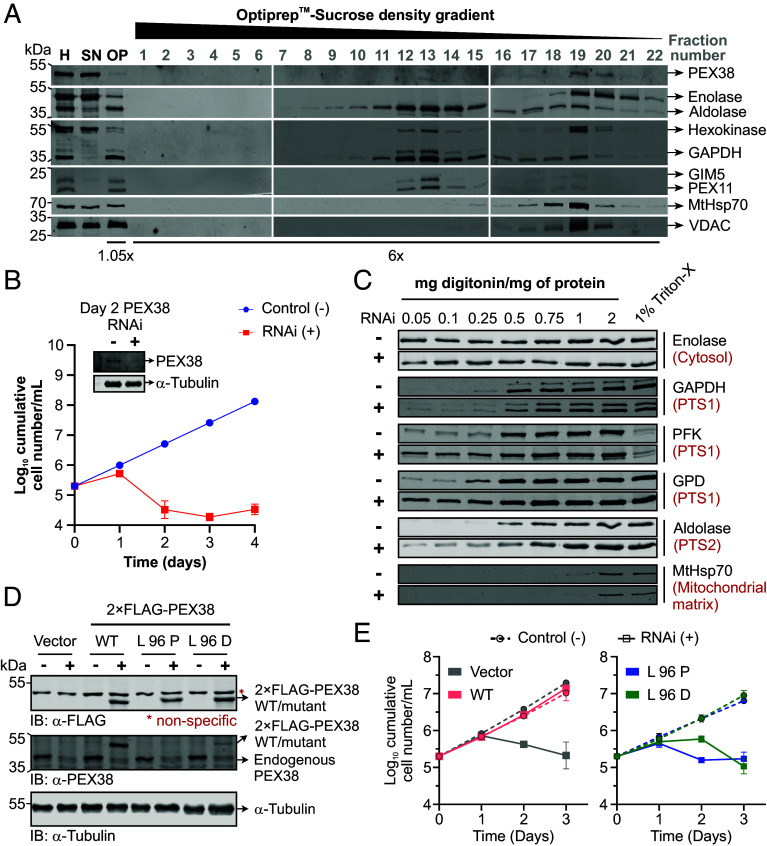
PEX38 is essential for glycosome biogenesis and parasite survival. (*A*) Subcellular fractionation of PCF trypanosomes by density gradient centrifugation. Fractions from the gradient bottom were analyzed by immunoblotting using subcellular markers. H, homogenate; SN, supernatant (cytosolic proteins); OP, organellar pellet. (*B*) Growth curve of BSF PEX38 RNAi cells ± RNAi induction (− DMSO, + tetracycline). Inset: Immunoblot showing PEX38 steady-state levels; α-tubulin, loading control. Error bars, SD; n = 3. (*C*) Biochemical fractionation of BSF PEX38 RNAi cells (day 2) with increasing digitonin concentrations. Supernatants analyzed by immunoblotting; 1% Triton X-100 included as a positive control. (*D*) Functional complementation of PEX38 RNAi cells by ectopic expression of 2× FLAG-tagged RNAi-resistant PEX38 (wild-type; WT, and mutants). Expression confirmed by immunoblotting. (*E*) Growth curves of PEX38 RNAi cells complemented with WT PEX38 (salmon), L96P (blue), or L96D (green) mutants, or empty vector (gray) ± RNAi induction. WT PEX38 restored growth upon RNAi induction, whereas L96P and L96D mutants and the empty vector exhibited growth defects. Error bars, SD; n = 3.

PEX38 lacks predicted TMDs or significant hydrophobic regions, suggesting that it is a soluble protein localized either in the cytosol or in the organelle matrix. To determine its subcellular localization, we performed subcellular fractionation of procyclic form (PCF) *T. brucei* cells. Cells were mechanically ruptured and subjected to differential centrifugation to separate the cytosolic fraction (supernatant) and the organelle-enriched pellet. The pellet was subsequently fractionated by density gradient centrifugation. Immunoblot analysis revealed that PEX38 was almost entirely released in the cytosolic supernatant of the initial differential centrifugation, like the cytosolic marker enolase, supporting the conclusion that PEX38 is primarily a cytosolic protein (Fig. 4*A*). A minor portion of PEX38 migrated to density gradient fractions 19 and 20, which contain mainly mitochondrial markers, but also trace amounts of enolase and glycosomal markers. Mature glycosomes migrate in fractions 12 to 14, while presence of glycosomal markers in fraction 19 may represent nascent glycosomes. In summary, PEX38 is predominantly cytosolic, with a small fraction potentially associating with immature glycosomes or other cellular compartments.

We also used *Trypanosoma* cell lines with ectopic, tetracycline-inducible N- or C-terminally GFP-tagged PEX38 to perform immunofluorescence microscopy and assess the localization of the GFP-tagged PEX38. The expression of these GFP-tagged constructs at correct predicted molecular mass was confirmed by immunoblotting (*SI Appendix*, Fig. S9*A*). Immunofluorescence microscopy was then performed using anti-aldolase antibodies as a glycosomal marker. GFP alone was used as a control to visualize cytosolic distribution. Neither of the overexpressed GFP-tagged PEX38 constructs colocalized with aldolase; instead, the overall cell labeling was like the GFP control, suggesting that the protein is localized in the cytosol (*SI Appendix*, Fig. S9*B*). However, while the overall cell labeling indicates cytosolic localization, the organelles are not excluded from the labeling, so an additional organellar localization cannot be distinguished by fluorescence microscopy.

The subcellular distribution of GFP tagged PEX38 was further examined biochemically by cellular fractionation. *T. brucei* cells expressing either N- or C-terminally GFP-tagged PEX38 were treated with a low concentration of digitonin, selectively permeabilizing the plasma membrane and releasing cytosolic contents into the supernatant. Enolase was used as a cytosolic marker, while aldolase served as a marker for glycosomes in the organelle-containing pellet. For both N- and C-terminally GFP-tagged PEX38, the majority of the proteins was detected in the cytosolic supernatant, in agreement with the microscopy data (*SI Appendix*, Fig. S9*C*). Notably, a small fraction of GFP-PEX38 was also detected in the organellar pellet (*SI Appendix*, Fig. S9*C*), suggesting a potential weak association with organelles, similar to what was observed for endogenous PEX38. However, the apparent higher amount of GFP-tagged PEX38 in pellet fraction may also result from overexpression.

### PEX38 and Its Interaction with PEX19 Is Essential for Glycosome Biogenesis and Parasite Survival.

To investigate the essentiality of PEX38 for *T. brucei*, we performed a tetracycline inducible RNA interference (RNAi) knockdown of PEX38 gene expression. Cumulative growth of RNAi induced or DMSO treated (negative control) bloodstream-form (BSF) as well as PCF parasites was monitored for 4 and 9 d, respectively. A severe growth defect is observed for both BSF as well as PCF trypanosomes (Fig. 4*B* and *SI Appendix*, Fig. S9*E*). Cell death was evident in the microscopic observation of parasite cultures. The depletion of PEX38 protein level was confirmed by immunoblot analysis (Fig. 4 *B*, *Insets*). The results demonstrate that PEX38 is critical for the parasite growth and viability of both BSF and PCF trypanosomes.

To investigate whether PEX38 depletion affects glycosome biogenesis, we performed immunofluorescence microscopy on *T. brucei* PEX38 RNAi cells (*SI Appendix*, Fig. S10). DMSO treated control cell lines showed a characteristic punctate pattern for the glycosomal marker enzymes GAPDH (PTS1 protein) and aldolase (PTS2 protein), consistent with proper glycosome localization. In contrast, RNAi-induced cells exhibited an abnormal glycosomes morphology and a partial mislocalization of both glycosomal matrix markers to the cytosol. However, due to the persistence of preexisting glycosomes, which stain brightly, the extent of mislocalization was difficult to assess solely by microscopy. To quantitatively evaluate this mislocalization, we conducted biochemical fractionation using digitonin permeabilization (Fig. 4*C*). Control or RNAi-induced cells treated with increasing concentration of digitonin were fractionated into cytosolic and pellet fractions. At low concentrations, digitonin permeabilizes the plasma membrane to release cytosol into the supernatant, as evidenced by the release of cytosolic marker enolase. Only at higher concentrations such as 0.5 mg digitonin/mg protein, organelles are permeabilized to release organellar matrix proteins. The analysis revealed that upon PEX38 RNAi, various PTS1- as well as PTS2-containing glycosomal enzymes are released into the cytosol at significantly lower concentrations of digitonin compared to the control. The mitochondrial matrix protein MtHsp70 was not affected by PEX38 RNAi. These findings demonstrate that PEX38 is required for proper glycosome biogenesis and matrix protein import in *T. brucei*.

Like PEX38, partial knockdown of PEX19 also results in a growth defect, demonstrating its essential role (21). To determine whether the parasite-specific PEX19–PEX38 PPI identified in this study is essential for parasite survival and thus a potential druggable target, we performed an in cellulo functional complementation analysis (22) in BSF *T. brucei* parasites (*SI Appendix*, Fig. S11*A*). We engineered a PEX38 RNAi cell line to express an ectopic, RNAi-resistant codon-exchanged PEX38 gene under the Tet-inducible promoter (*SI Appendix*, Fig. S11 *A* and *B*). Tet-induction of this double-transfected cell line results in depletion of the endogenous PEX38 and simultaneous expression of the ectopic RNAi-resistant PEX38, which was Flag-tagged to distinguish it from endogenous PEX38. Two controls were used in this assay: a negative control transfected with an empty vector, and a positive control transfected with nonmutated PEX38. Guided by our structural characterization, we chose the conserved L96 residue within the Ecd/SGT1 domain of PEX38, which mediates contact with PEX19, for site-directed mutagenesis. We generated two mutants: L96P (disrupting helix formation) and L96D (disrupting hydrophobic interactions) and tested them in the same complementation assay. Prior to growth analysis, immunoblotting analysis confirmed the expression of all ectopic constructs using an anti-Flag antibody (Fig. 4 *D*, *Upper* panel) and efficient knock-down of endogenous PEX38 with anti-PEX38 antibodies (Fig. 4 *D*, *Middle* panel).The mutant constructs (L96P/D) were clearly detected with Flag-antibodies, but either faintly or not detected by the PEX38 antibody, suggesting these mutations may induce structural changes that compromise antibody recognition. Upon tetracycline induction, the negative control displayed growth defect from PEX38 RNAi, while expression of wild-type PEX38 fully rescued the phenotype, restoring near-normal growth (Fig. 4 *E*, *Left* panel). In contrast, neither the L96P nor L96D mutants were able to rescue the parasite growth (Fig. 4 *E*, *Right* panel). Taken together, these results demonstrate that the PEX38–PEX19 PPI is essential for *Trypanosoma* parasite survival

PEX38 orthologs could be identified in all kinetoplastids as well as in *Diplonema*, which together belong to phylum Euglenozoa. As PEX38 appears to be specific to Euglenozoa, we extended our study to other clinically relevant kinetoplastids. Multiple sequence alignment of PEX38 orthologs from various *Trypanosoma* and *Leishmania* species and *Diplonema* reveals that the PEX19 binding region in PEX38 is highly conserved (*SI Appendix*, Fig. S12*A*). Notably, the residues L96 and L99, which mediate contacts with PEX19, are conserved across species (*SI Appendix*, Fig. S12*A*). Overall, these sequences share at least 55% identity and more than 63% similarity with *Tb*PEX38 (*SI Appendix*, Fig. S12*B*). To test whether this interaction is functionally conserved, we performed Y2H assays using *L. donovani* PEX19 (*Ld*PEX19) and PEX38 (*Ld*PEX38). As expected, *Ld*PEX19 interacted with wild-type *Ld*PEX38. Importantly, proline substitutions corresponding to the L96 and L99 residues of *Tb*PEX38 abolished the interaction with *Ld*PEX19 (*SI Appendix*, Fig. S12*C*), emphasizing the importance of these residues in the clinically relevant organism *L. donovani*. Taken together, these findings demonstrate that the PEX19–PEX38 PPI is conserved across trypanosomatid species. Given its functional importance and parasite specificity, the PEX19–PEX38 interaction represents a promising drug target for the development of new therapeutics against trypanosomatid infections.

### PEX38 Bridges the PMP Import Receptor PEX19 with the Protein Folding Machinery via Hip.

**Fig. 5.**
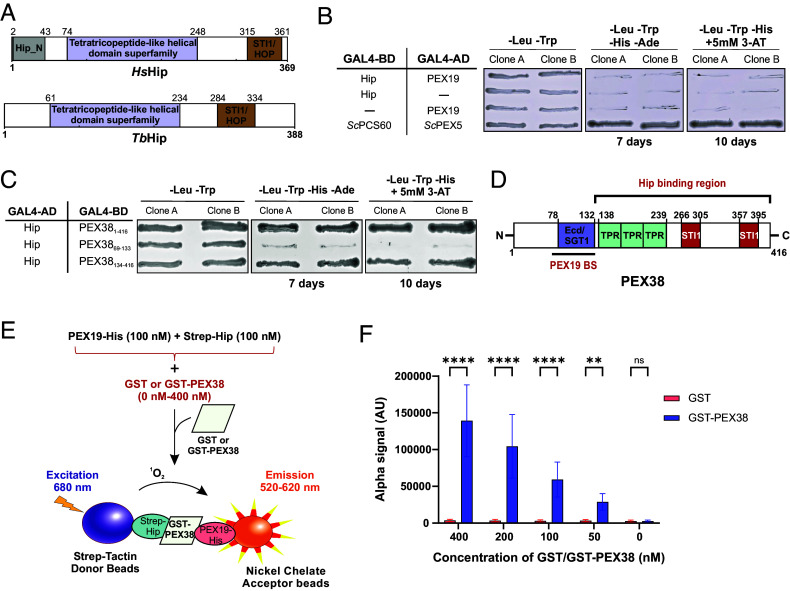
PEX38 connects PEX19 to the chaperone machinery via Hip. (*A*) Domain architecture of *Hs*Hip and *Tb*Hip. (*B*) Y2H assay testing PEX19–Hip interaction (second most prominent hit in PEX19 interactome; Fig. 1*B*). Growth-based assay with two independent yeast clones streaked on selective media: −Leu −Trp (growth control), −Leu −Trp −His −Ade (7 d), and −Leu −Trp −His + 5 mM 3-AT (10 d). *Sc*PEX5-*Sc*PCS60, positive control; no autoactivation, negative control. (*C*) Y2H analysis of Hip-PEX38 (FL and variants) interaction. Growth-based assay with two independent yeast clones streaked on selective media as in panel (*B*) (negative controls, *SI Appendix*, Fig. S13*A*). (*D*) Schematic summary of PEX19 and Hip binding to PEX38, showing distinct interaction regions identified in Figs. 2*B* and 5*C*). (*E*) Schematic of AlphaScreen binding assay demonstrating PEX38-mediated bridging of PEX19–Hip ternary complex formation. (*F*) AlphaScreen assay with 100 nM PEX19-His and Strep-Hip with varying concentrations of GST-PEX38 or GST. Statistical comparison by two-way ANOVA with the Šídák test: ****P < 0.0001; **P = 0.0013; ns, not significant. Error bars, SD; n = 3 independent experiments, 6 technical replicates each.

Previous studies suggested that PEX19 acts as a cytosolic chaperone for newly synthesized PMPs (5). Overexpressed PEX19 has been demonstrated to stabilize PMPs, while its absence leads to PMP aggregation (5, 23–25). Despite this, the mechanism by which PEX19 mediates PMP chaperoning remains unclear. Along this line, the *Leishmania* ortholog of PEX38, originally termed *Ld*SGT, has been characterized as a cochaperone that forms a stable complex with the Hip and associates with Hsp70 and Hsp90 chaperones (26). To investigate this further, we examined the *Tb*PEX19 interactome in detail and identified a putative Hip homolog as one of the top interactors. Particularly, like PEX38, the putative Hip protein was absent in affinity pull-downs from cytosolic fraction with PEX19_ΔN30_ as bait, indicating that its interaction is dependent on the same PEX19 region (Fig. 1*B*). This protein retrieves the human Hip as the top hit in BLAST search. Henceforth, we will refer to it as the *Trypanosoma* Hip (*Tb*Hip). Domain analysis revealed that *Tb*Hip shares key features with the human Hip, including conserved TPR-like helical and STI1/HOP domains (Fig. 5*A*). However, unlike its human counterpart, *Tb*Hip lacks the N-terminal dimerization domain (Hip_N), suggesting potential differences in complex assembly or regulation.

To further validate the proteomic identification of Hip as PEX19 interactor, we investigated the interaction between PEX19 and Hip using Y2H assays. Unlike PEX38, *Tb*Hip did not interact with PEX19 in the Y2H assay (Fig. 5*B*), suggesting that the association observed in the PEX19 interactome may be indirect. We next tested whether *Tb*Hip interacts with PEX38. All proteins/truncations tested here were verified not to exhibit autoactivation (*SI Appendix*, Fig. S13*A*). Full-length PEX38, as well as truncation constructs spanning residues 134 to 416 that completely lack PEX19 binding site, shows clear interaction with *Tb*Hip. In contrast, PEX38 variants encompassing the PEX19 binding regions (residues 69 to 133 comprising BS1–BS3) did not interact with Hip (Fig. 5*C*). These findings suggest that Hip interacts with the C-terminal region of PEX38 (residues 134 to 416), independent of the PEX19-binding region (Fig. 5*D*). Thus, the detection of Hip in the PEX19 interactome is likely mediated by its interaction with PEX38 rather than by a direct association with PEX19. The C-terminal region of PEX38 (residues 134 to 416), which mediates interaction with Hip, contains two STI1 domains and a region distinct from the PEX19 binding region comprising the Ecd/SGT1 domain. This domain separation may suggest that PEX38 can simultaneously bind to both PEX19 and Hip via nonoverlapping regions.

To assess whether disruption of the PEX19-binding site affects PEX38’s ability to bind Hip, we tested PEX38 mutations that disrupt PEX19 binding using a Y2H assay. Both wild-type as well as the mutants (mutations L96 to P or D and LDL 96 to 98 to PPP), showed similar levels of interaction with Hip (*SI Appendix*, Fig. S13*B*). These findings indicate that mutations impairing the binding of PEX38 to PEX19 do not interfere with Hip binding, further supporting the notion that PEX38 has separable binding interfaces for these two partners.

To directly test whether PEX38 can bridge PEX19 and Hip into a ternary complex, we performed an AlphaScreen interaction assay using recombinant proteins: PEX19-His, GST-PEX38, and Strep-tagged Hip, with GST also as a control. PEX19-His was preincubated with increasing amounts of GST or GST-PEX38, followed by the addition of Strep-Hip. A robust increase in the AlphaScreen signal was observed with increasing amounts of GST-PEX38, indicating the formation of a ternary complex, involving PEX19, PEX38, and Hip (Fig. 5 *E* and *F*). In contrast, no significant signal was detected between PEX19 and Hip in absence of PEX38 or in the presence of GST alone, consistent with the Y2H study (Fig. 5*B*). These data strongly suggest that PEX38 bridges the interaction of PEX19 and Hip to form a ternary complex. This complex may functionally link the targeting of peroxisomal membrane proteins by PEX19 with their folding of stabilization via the chaperone-associated cofactor Hip, highlighting a coordinated mechanism for glycosomal membrane protein biogenesis.

## Discussion

The mechanism by which PEX19 chaperones and targets PMPs, containing single or multiple TMDs and inserts them in their correct topology into the glycosomal membrane is poorly understood. An earlier study reported that the C-terminal domain of PEX19, although capable of binding PMPs, is insufficient for maintaining the solubility of in vitro synthesized PMPs (25). Moreover, combining separately purified N- and C-terminal domains of PEX19 also failed to stabilize these proteins (25). A recent study showed that chaperoning of a mammalian TA protein PEX26 by PEX19 involves the amphipathic helix in the C-terminal domain of PEX19, while insertion requires another amphipathic helix in the N-terminal domain (27). These observations suggested that PEX19 N-terminal domain has additional functions apart from binding to PEX3. Here, we report on the identification and characterization of PEX38, a kinetoplastids-specific peroxin as a key cytosolic bridging factor that links the PMP import receptor PEX19 to the cytosolic chaperone machinery via Hip, a cochaperone.

In most eukaryotes, PEX19 contains a C-terminal CaaX motif that undergoes farnesylation, enhancing its affinity for PMPs (28). However, euglenozoan PEX19 proteins, including those in *Trypanosoma* and *Leishmania*, lack the CaaX motif and are not farnesylated. Interestingly, the *Leishmania* ortholog of *Tb*PEX38 (previously termed *Ld*SGT) was shown to act as a cochaperone, forming stable complexes with the chaperone machinery, including Hsp70, Hsp90, and the cochaperone Hip (26). In our study, proteomic analysis identified Hip and Hsp70 along with PEX38 in the *Trypanosoma* PEX19 interactome, which were significantly reduced in the interactome upon deletion of the PEX38 binding motif, suggesting that PEX38 serves as a mediator for chaperone recruitment to PEX19. This mechanism may compensate for the absence of farnesylation in *Trypanosoma* PEX19 or represent an alternative strategy to engage chaperones during PMP import, since farnesylation is known to allosterically enhance PEX19’s recognition of PMPs by reshaping its binding surface (28). A similar mechanism has been reported to occur in mitochondria, where receptors TOM22 or TOM70 selectively recognize cytosolic Hsp70 cochaperones that bind hydrophobic precursor proteins and assist in their transfer from the cytosol to the mitochondrial receptors (29).

The N-terminal region of PEX38 contains a conserved domain homologous to the Ecd/hSGT1 domain, previously recognized for its role in cell cycle progression in mammals (30). We demonstrate that this region specifically mediates binding to the N-terminal PEX3-binding motif of PEX19 in both *Trypanosoma* as well as *Leishmania*.

**Fig. 6.**
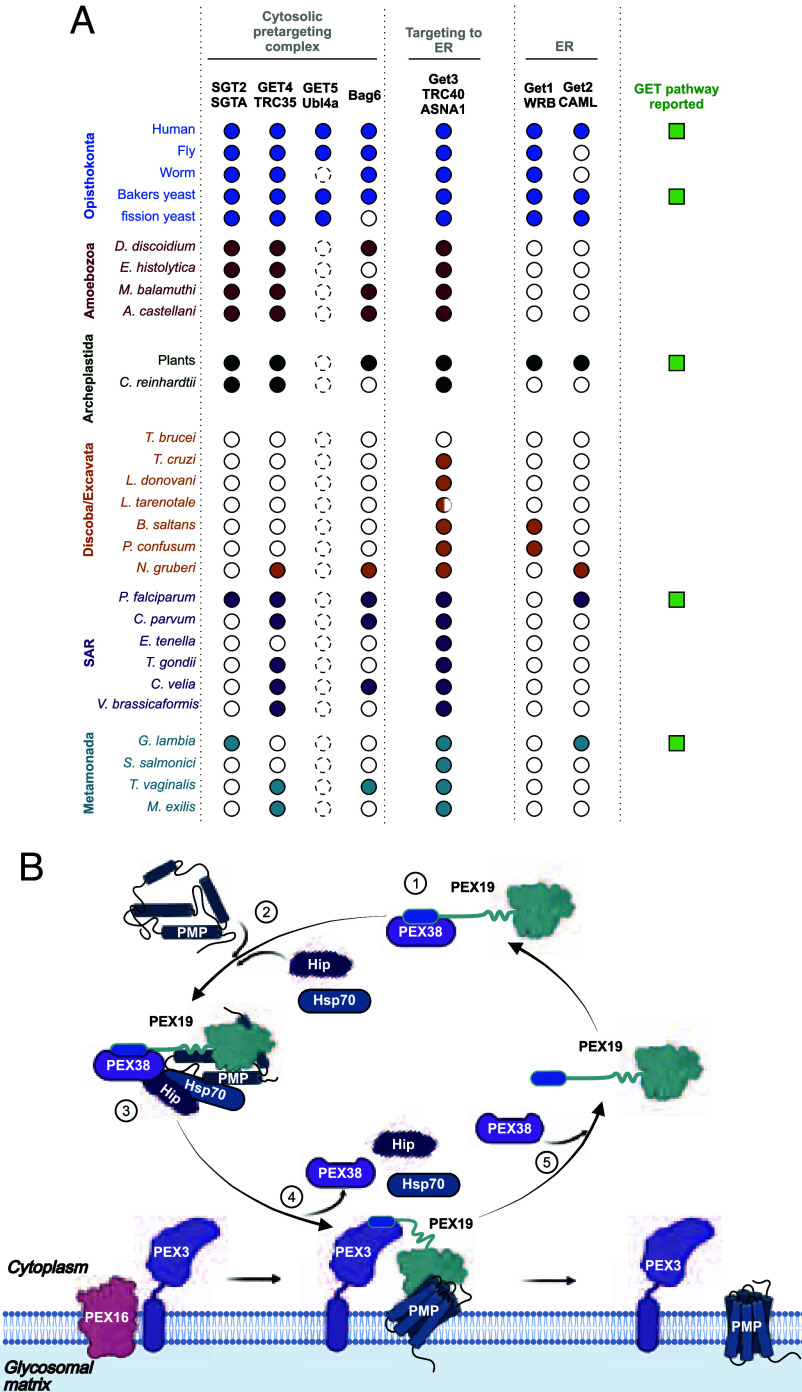
Evolutionary analysis and mechanistic model of glycosomal membrane biogenesis. (*A*) Coulson plot depicting conservation of GET/TRC pathway orthologs across organisms. Yeast and human proteins were used to identify orthologs in other organisms. Dotted circles, multiple candidates not unambiguously assigned to Get5/Ubl4a; half-colored circles, identified partial fragments. (*B*) Proposed model of glycosomal membrane protein (PMP) import: 1) Receptor priming: PEX38 binds PEX19, preventing premature docking without cargo. 2) Chaperone-mediated stabilization: newly synthesized PMPs with mPTS signals are stabilized by Hip/Hsp70 chaperones. 3) Predocking complex formation: PEX19–PEX38 recognizes the mPTS signal of PMP, which are associated with chaperones. PEX19–PEX38 with folded PMP is ready for docking. 4) Docking and insertion: PEX19–PMP complex docks to membrane-anchored PEX3. During this step, PEX38 is displaced by PEX3 due to its higher affinity for PEX19 (handover mechanism), allowing PMP insertion into the membrane. PEX38 displacement accompanies dissociation of Hip and chaperones. Chaperones facilitate active unfolding and folding of PMPs during membrane insertion. 5) PEX19 recycling: PEX19 dissociates and rebinds PEX38 in the cytosol, for the next import cycle.

PEX38 shares partial structural similarity with SGT2/SGTA family of cochaperones from the Guided Entry of TA proteins (GET in yeast)/transmembrane recognition complex (TRC in humans) pathway, a conserved mechanism of targeting TA proteins to the ER (31), as recognized earlier (26). SGT proteins, such as Sgt2 in yeast and SGTA in humans capture newly synthesized TA proteins in the cytosol and target them to the ER membrane, acting in conjunction with other GET proteins or their homologs (reviewed in (32, 33)). However, PEX38 lacks key SGT features, including the conserved SGTA N-terminal homodimerization domain and the C-terminal glutamine-rich region. This, along with its unique domain architecture and demonstrated role in glycosome biogenesis, supports PEX38’s classification as a peroxin rather than a canonical SGT protein. The GET/TRC pathway has been studied mainly in fungi and metazoans, and it is also present in plants and lower eukaryotes, including the human pathogens Plasmodium and *Giardia* (34). Interestingly, no clear homologs of canonical GET/TRC pathway components, including SGT2/SGTA, were identified in *T. brucei*. A putative Get1 homolog was detected in *Bodo saltans* (*SI Appendix*, Fig. S14*A*), and a Get3 homolog is present in *B. saltans, Trypanosoma cruzi,* and several *Leishmania* species (*SI Appendix*, Fig. S14 *B* and *C*), although certain species such as *L. tarentolae* encode only fragmented versions. Our bioinformatic analysis (Fig. 6*A*) suggests that while the GET/TRC pathway was likely present in the common ancestor of kinetoplastids, it has been partially or fully lost in several lineages. In this context, PEX38 may represent a lineage-specific adaptation in Euglenozoa, functionally replacing components of the GET/TRC-system to facilitate chaperoning of PMPs in the absence of canonical machinery. This finding aligns with the emerging view that gene loss plays an important role in diversification of protein complexes and enhancement of eukaryotic diversity (35).

Depletion of PEX38 resulted in glycosome biogenesis defects and parasite death, confirming its essential role as peroxin (16, 36). Structurally, our study revealed that the binding region of PEX19 for PEX38 overlaps with its PEX3 binding motif, which is crucial for docking PEX19–PMP complexes at the glycosomal membrane (16, 18). Accordingly, our displacement assay revealed a competition between PEX3 and PEX38 for binding to PEX19, but PEX3 binds this region with ~30-fold higher affinity than PEX38 in vitro. However, in vivo conditions favor dynamic competition: PEX38 is more abundant in the cytosol than membrane-anchored PEX3 and therefore more likely associated with cytosolic PEX19. PEX3 or PEX38 binding to PEX19 would be dynamic in vivo depending on the stage of PMP import cycle and the competition might even serve as functional cooperation. Thus, the interplay between PEX3 and PEX38 may represent a regulated handover mechanism during different stages of PMP import, akin to competitive/cooperative interactions seen in other trafficking pathways (e.g., Inp1 vs. PEX19 for PEX3) (37).

The PEX3 binding motif of PEX19 is intrinsically disordered in the absence of PEX3 and is assumed to adopt an α-helical conformation when bound to PEX3 (10). Our NMR analysis demonstrates that PEX38 also induces disorder-to-helix transition in PEX19. Proteins with intrinsically disordered regions play key roles in protein networks, enabling communication through PPIs. In fact, large disordered regions play key roles in peroxisome biogenesis and have been identified in PEX19 homologs but are also found in the N-terminal region of the cargo receptor PEX5 and in the docking protein PEX13 (38–40). Many regulatory proteins have disordered motifs that fold when binding cellular targets, though the underlying mechanisms are unclear (41).

Based on our findings, we propose a working model for the glycosomal membrane protein import in which PEX38 bound PEX19 interacts with newly synthesized PMPs that are thereby stabilized by Hip and associated chaperones to ensure the proper folding and delivery to the glycosomal membrane (Fig. 6*B*). At the peroxisomal membrane, competition of PEX3 for PEX19 binding will result in the handover of the PMP-loaded PEX19 to PEX3 and in the release of PEX38 together with the associated chaperone machinery. Experimental validation of this model will be critical for understanding the full mechanistic cycle of glycosomal PMP import. Nonetheless, parallels can be drawn with the recently identified PEX39. In an initial step, PEX39 associates with the PTS2 receptor PEX7 via its KPWE motif, followed by cargo protein and coreceptor binding. The resulting complex then docks at the peroxisomal membrane by binding of PEX7 to the KPWE motif of PEX13, leading to displacement of PEX39 via a handover mechanism (42). In addition, the KPWE motif of PEX39 has been implicated in the final step of PTS2 protein import, where it promotes extraction of PEX7 from the PEX13 YG phase and primes the receptor for another round of import cycle (43).

Importantly, glycosomes are a therapeutic vulnerability (designated as Achile’s heel) of trypanosomatid parasites and represent a validated drug target class for the development of new therapies for devastating trypanosomiasis and leishmaniasis. PEX38 is essential, kinetoplastid-specific, with no equivalent human counterpart. Using in vivo functional complementation, we further validated that disrupting the PEX38–PEX19 interaction is lethal to the parasites. Combined with our structural insights, these findings nominate the PEX38–PEX19 binding interface as a promising molecular target for structure-based drug discovery against trypanosomiasis and leishmaniasis.

## Methods

### Molecular Biology, Protein Expression, and Purification.

Expression constructs for *E. coli*, yeast, and *T. brucei* used in biochemical assays, Y2H, RNAi, complementation, and structural studies were generated by standard cloning (*SI Appendix*, Tables S4-S5) and verified by Sanger sequencing. Recombinant GST-, His-, or Strep-tagged PEX19, PEX38, PEX3, Hip, and control proteins were expressed in *E. coli* with IPTG induction and purified by affinity chromatography. Detailed protocols are provided in the *SI Appendix*, *Methods*.

### Proteomics and MS.

PEX19 interaction partners were enriched by affinity pulldown from cytosol- and organelle-enriched *T. brucei* fractions prepared by digitonin-based permeabilization, separated by SDS-PAGE, subjected to in-gel tryptic digestion, and analyzed by nanoLC-ESI-MS/MS with subsequent computational data processing. Detailed protocols for pulldown, in-gel digestion, LC-MS/MS acquisition, and data analysis are provided in the *SI Appendix*, *Methods*.

### Biophysical and Structural Analysis.

Biophysical characterization, including NMR spectroscopy, ITC, and AlphaScreen assays, was used to map binding sites, define complex formation, and determine binding affinities. Experimental conditions for isotope labeling and sample preparation, NMR data acquisition and structure calculation, ITC measurements and fitting, and AlphaScreen binding and competition formats are detailed in the *SI Appendix*, *Methods*.

### *Trypanosoma* Cell Culture and Cell-Based Assays.

*Trypanosoma brucei* bloodstream and procyclic forms were cultured under standard conditions, genetically engineered by stable transfection for inducible expression and RNAi, and analyzed by subcellular fractionation, complementation, and fluorescence microscopy. Detailed information on cell culture conditions, transfection and RNAi protocols, density gradient and digitonin-based fractionation, complementation assays, microscopy and image analysis settings, cDNA synthesis, and immunoblotting procedures is provided in the *SI Appendix*, *Methods*.

### Yeast Two-Hybrid, Bioinformatics, and Statistical Analysis.

Y2H assays were performed using established *Saccharomyces cerevisiae* reporter strains to validate PPIs. Orthologs and domain architectures were identified using standard bioinformatics tools (domain prediction, sequence alignment, and structural similarity searches). Immunoblotting employed organellar and cytosolic markers with custom anti-PEX38 antibodies. Proteomics and AlphaScreen data were analyzed using false-discovery-rate controlled protein identification, intensity-based quantitation, data imputation and normalization, ANOVA, and nonlinear regression for IC_50_ estimation. Detailed protocols are provided in the *SI Appendix*, *Methods*.

## Supplementary Material

Appendix 01 (PDF)

Dataset S01 (XLSX)

## Data Availability

NMR data have been deposited in wwPDB under deposition number D_1292150397 with accession code PDB ID: 9SGX and will be publicly available upon publication. MS proteomics data have been deposited in the ProteomeXchange Consortium (44) via the PRIDE partner repository (dataset identifier PXD068882) and will be publicly available upon publication. The analysis pipeline and statistical tools are documented in a Jupyter notebook hosted at https://github.com/ag-warscheid/Tb_PEX38_manuscript, which will be publicly accessible upon publication.
