# Supplementary material for: Evolutionary remodeling of a remnant GET pathway factor into PEX38, an essential peroxin
Source: Proc Natl Acad Sci U S A. 2026 Feb 26;123(9):e2533726123. doi: 10.1073/pnas.2533726123 (PMC12956874; doi:10.1073/pnas.2533726123)
Supplement: Supplementary file 1 — Appendix 01 (PDF) [file pnas.2533726123.sapp.pdf]

PNAS

Supporting Information for

Evolutionary remodelling of a remnant GET pathway factor into PEX38, an essential peroxin

Chethan K. Krishna[a], Stefan Gaussmann[b,c], Hirak Das[d], Martin Jung[e], Silke Oeljeklaus[d], Michael Sattler[b,c], Bettina Warscheid[d], Vishal C. Kalel[a,*], Ralf Erdmann[a,*]

*To whom correspondence may be addressed
Email: ralf.erdmann@ruhr-uni-bochum.de,
vishal.kalel@ruhr-uni-bochum.de

This PDF file includes:

Figures S1 to S14
Tables S1 to S5
Legends for Dataset S1
SI Methods
SI References

Figures

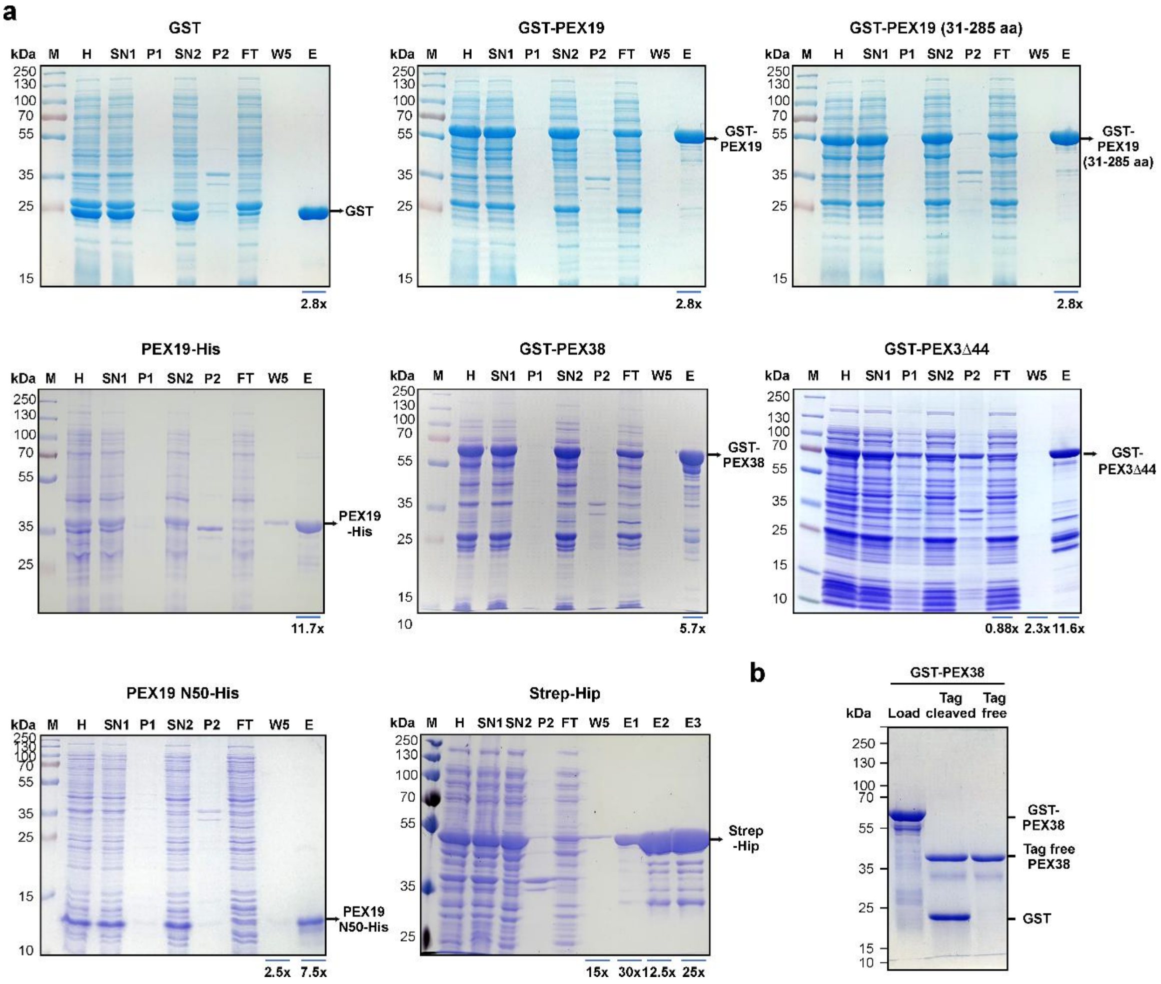

Fig. S1. Affinity purification profiles of the recombinant proteins. a) Various bacterially expressed proteins were affinity-purified using Glutathione-, Ni-NTA- or Strep-Tactin-XT- Agarose Beads according to the tag as indicated. Samples were collected at each step and analysed by SDS-PAGE and Coomassie staining. The samples include H, Homogenate; SN1, supernatant 1; P1, Pellet 1; SN2, supernatant 2; P1, Pellet 2; FT, flow-through; W5, Wash 5; E, Elution. **b)** Preparation of tag-free PEX38. The GST-tag was cleaved from GST-PEX38 using biotinylated thrombin, which was then captured using streptavidin beads, while the cleaved GST-tag was subsequently removed using glutathione agarose beads.

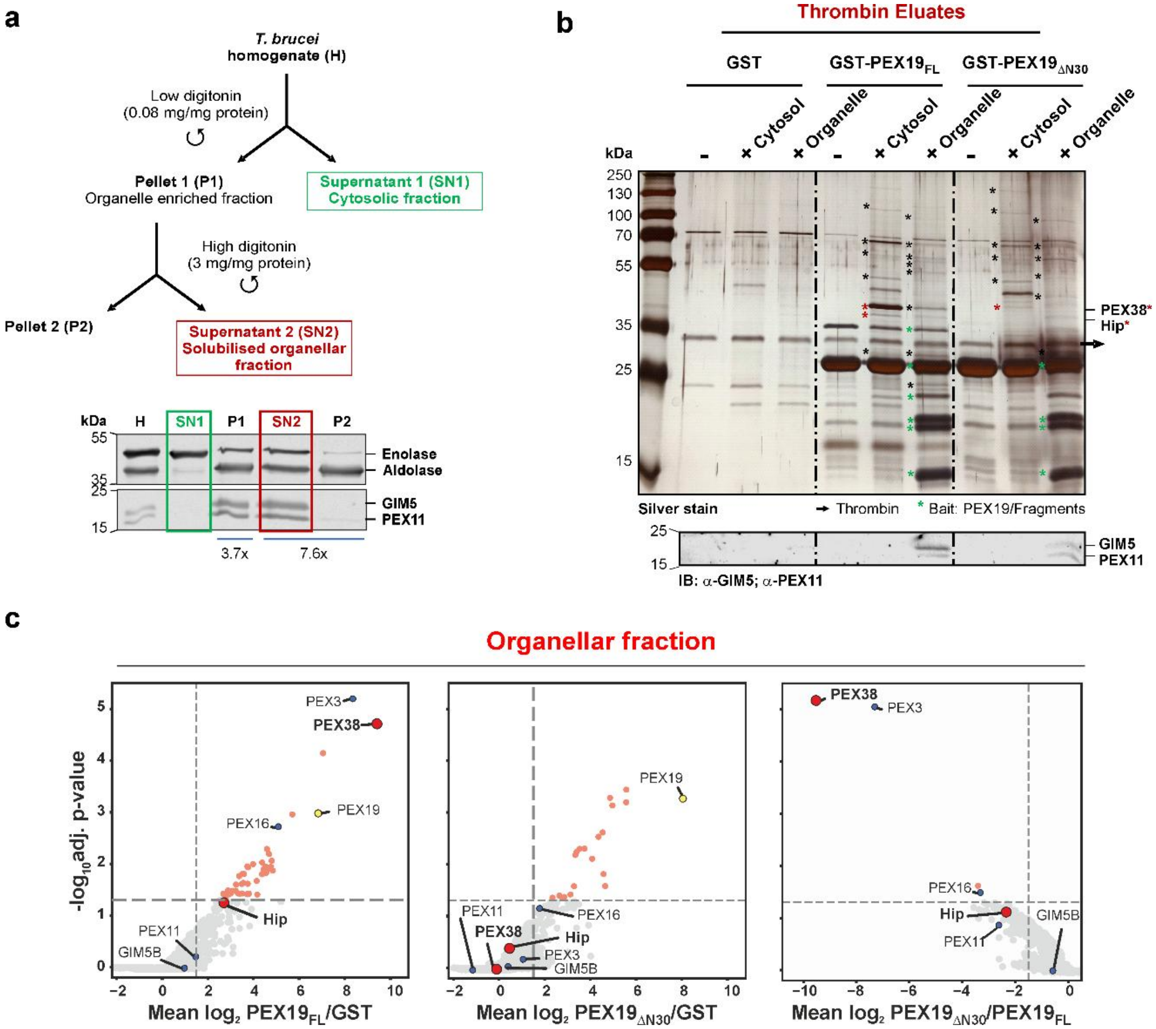

Fig. S2. Identification of PEX19-interacting proteins from cytosolic and organellar fractions of trypanosomes: a) Scheme for the preparation of cytosol and organelle-rich fractions from procyclic form *Trypanosoma brucei* cells (upper panel). Samples collected at each step of the preparation were analysed by SDS-PAGE followed by immunoblotting with polyclonal antibodies against *T. brucei* enolase, aldolase, GIM5, and PEX11. **b)** Cytosolic and organellar binding partners of *T. brucei* PEX19 were isolated using affinity pull-down using GST alone as control or GST-tagged PEX19 full-length ($PEX19_{FL}$) or a PEX19 variant lacking the N-terminal 30 amino acids ($PEX19_{\Delta N30}$). The thrombin-eluted fractions were analysed by SDS-PAGE and silver staining. Putative binding partners of PEX19 that are visible as additional bands in both cytosolic and organelle-enriched fractions but not in the control GST are marked with black asterisks. Lower panel: Immunoblot analysis using polyclonal antibodies against GIM5 and PEX11. **c)** Affinity Purification Mass Spectrometry (AP-MS) analysis of the PEX19 (FL and ΔN30) interactomes from trypanosomal organellar fractions. Ratios (left to right) of $PEX19_{FL}$/GST, $PEX19_{\Delta N30}$/GST , and $PEX19_{\Delta N30}$/ $PEX19_{FL}$ were calculated from quantified MS-based iBAQ, and a rank-product test was performed for the identification of specifically bound proteins and determination of adjusted p-values (1). Highlighted proteins are bait PEX19 (yellow), known glycosomal membrane proteins (blue), PEX38, and HiP (red) and proteins with significant fold change (light red). Vertical dashed lines indicate a $\log_2$ ratio of greater than or less than 1.5 (left, middle) or less than 1.5 (right), respectively ($n \geq 2$ replicates), and the horizontal line indicates a false discovery rate of 5% ($n = 3$ biological replicates).

a

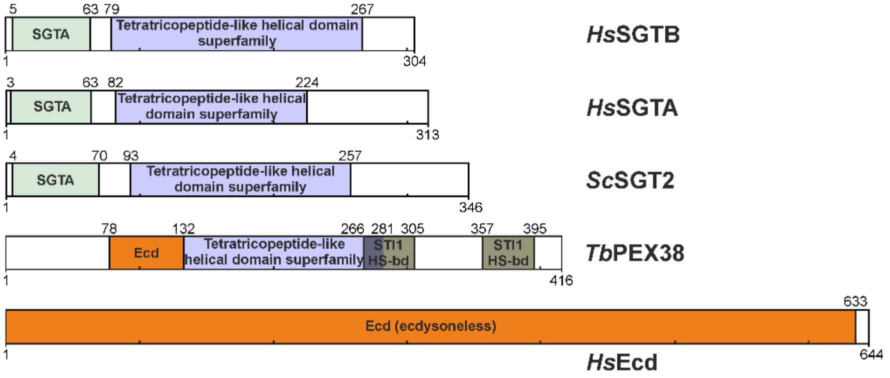

b

	Tb PEX38	*Sc* SGT2	*Hs* SGTA	*Hs* SGTB	% Similarity
Tb PEX38		34.68	40.57	39.14	
Sc SGT2	23.98		39.93	37.5	
Hs SGTA	30.67	32.58		67.43	
Hs SGTB	28.61	28.61	58.22		
	% Identity				

c

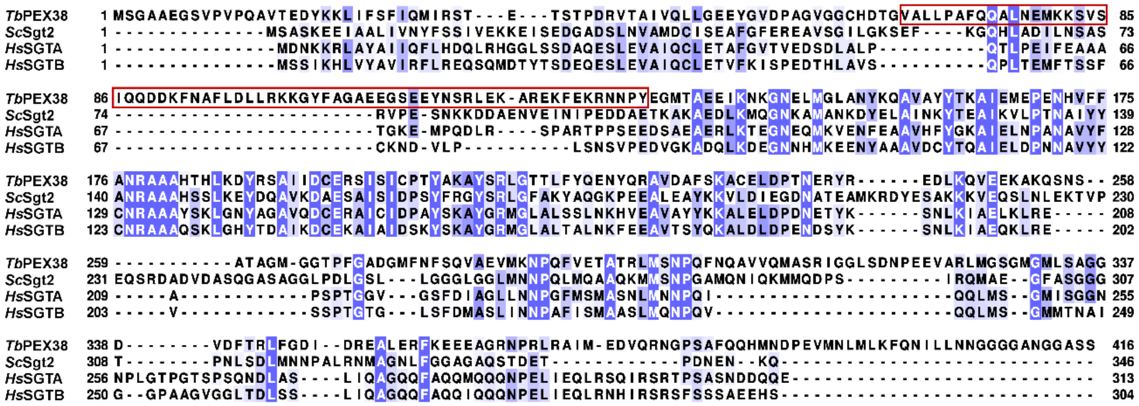

Fig. S3. Bioinformatic analysis of PEX38. a) Domain architecture of PEX38, human and yeast SGT family proteins and the human Ecd protein. SGTA, SGTA homodimerisation domain; STI1 HS-bd, STress Inducible 1 Heat Shock chaperonin binding domain; Ecd, ecdysoneless, identified using the InterPro domain database. **b)** The percentage identity and similarity matrix of PEX38 with the SGTA or SGTB protein of human and yeast counterparts, generated using SIAS homology modelling. **c)** Multiple sequence alignment of *Sc*Sgt2, *Hs*SGTA/B, and *Tb*PEX38 protein sequences. The sequence conservation is coloured according to the percentage identity with a conservation threshold of 30%. The identified PEX19 binding region within the PEX38 of *T. brucei* is indicated by the red box.

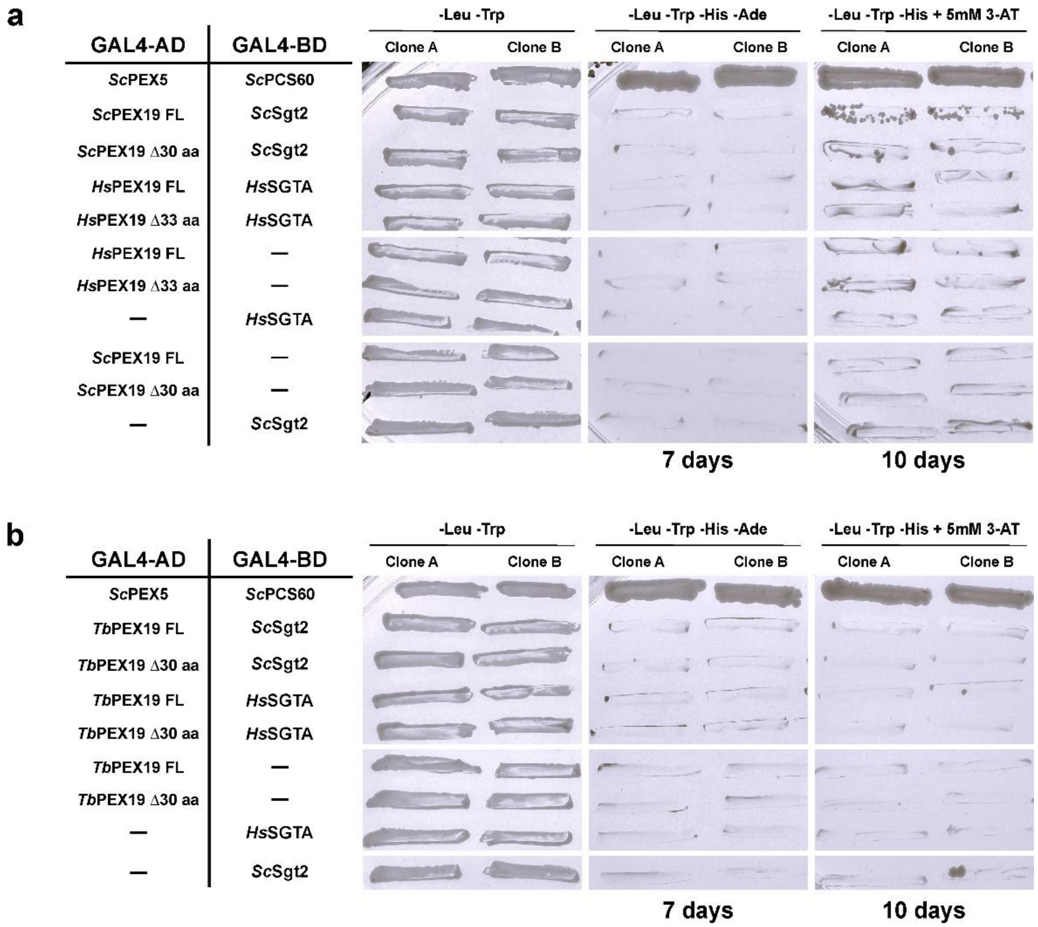

Fig. S4. Y2H analysis of Human *(Hs)*, *S. cerevisiae (Sc)*, and *T. brucei (Tb)* PEX19 interactions with SGTA and Sgt2. Y2H assays were performed in the *S. cerevisiae* PJ69-4A strain using constructs fused to either the GAL4 activation domain (AD) or DNA-binding domain (BD). **a)** Interactions were tested between full-length (FL) PEX19 and a PEX19 variant lacking the PEX3-binding motif (Δ30aa) with SGTA and Sgt2 from *Hs* and *Sc*, respectively. No interaction was detected between *Hs*PEX19 (FL and Δ30aa) and *Hs*SGTA, except for a very weak interaction observed between *Sc*PEX19 FL and *Sc*Sgt2. **b)** Interactions were also tested between *T. brucei* PEX19 (*Tb*PEX19 FL and Δ30aa) and *Hs*SGTA or *Sc*Sgt2. No interactions were observed for *Tb*PEX19 with SGTA or Sgt2. *Sc*PEX5 and *Sc*PCS60 served as a positive control, while the negative control showed no autoactivation.

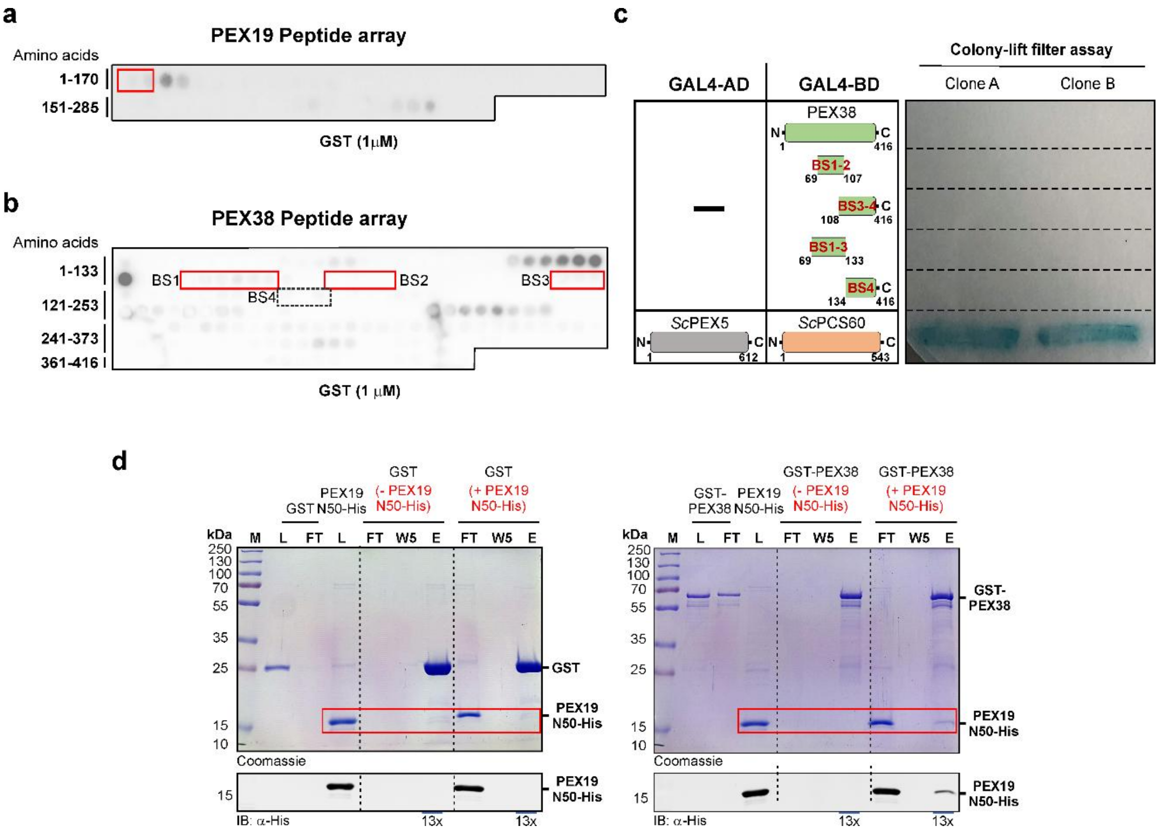

Fig. S5. a, b) The PEX19 and PEX38 peptide arrays were probed with GST-PEX38 and GST-PEX19 (as shown in **Fig. 2a** and **2b**) as well as with the control protein GST (as shown here), which served as a negative control. Red and black boxes indicate regions with no background signal, while binding was observed with the test proteins. **c)** The GAL4-AD fusion of PEX19 and the various GAL4-BD fusions of PEX38 were tested for autoactivation in Y2H assay. No colour development was observed in the assay, indicating that the constructs do not exhibit autoactivation. ScPEX5-PCS60 served as a positive control for the study (as shown in **Fig. 2c**). **d)** A pull-down assay was performed in vitro using recombinant GST-PEX38 (right panel) or GST (left panel, negative control), which were pre-incubated with glutathione agarose beads. This was followed by incubation with the PEX19 N50-His protein. The lanes correspond to L (load), FT (flow-through), W5 (wash 5), and E (eluate) for each condition indicated above the lanes. Bound proteins were eluted with reduced glutathione and analyzed by SDS-PAGE and Coomassie Blue staining. The PEX19 N50-His protein was found to be pulled down with GST-PEX38, which was further confirmed by immunoblotting using an anti-His monoclonal antibody (lower panel).

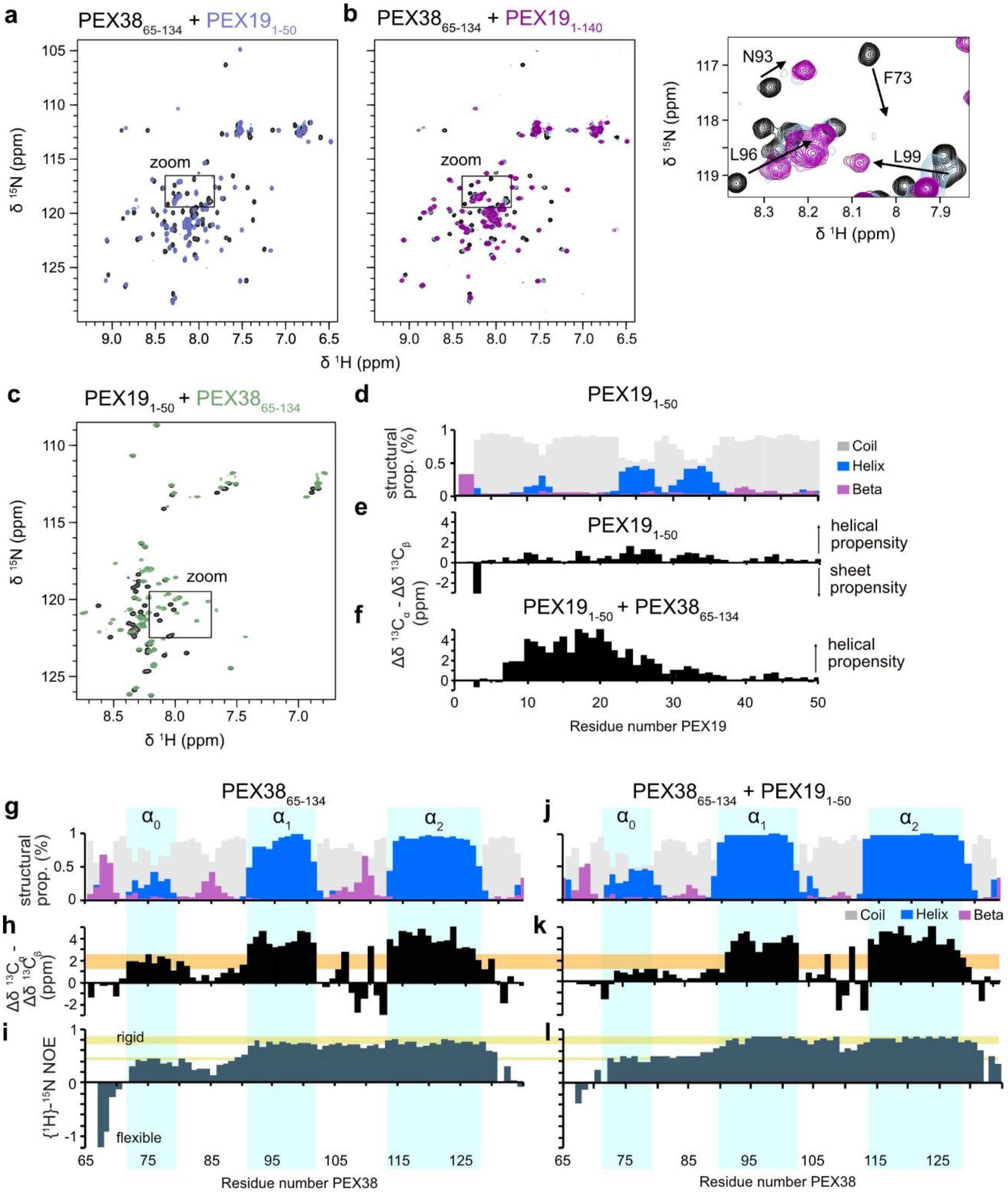

Fig. S6. Binding of PEX19 to PEX 38 induces a helix in PEX19 and stabilizes the helical bundle in PEX38. Overlaid ^{1}H-^{15}N spectra of ^{15}N labeled PEX38$_{65\text{-}134}$ (black) titrated with increasing concentrations of **(a)** PEX19$_{1\text{-}50}$ (purple scale; zoom is shown in **Fig. 3b**) and **(b)** PEX19$_{1\text{-}140}$ (magenta scale) with zoom shown on the right. **c)** Overlay of ^{1}H-^{15}N spectra of PEX19$_{1\text{-}50}$ (black) and PEX19$_{1\text{-}50}$ saturated with PEX38$_{65\text{-}134}$ (green) (zoomed region is shown in **Fig. 3a**). **d)** TALOS-N, Secondary structure propensity of free PEX19$_{1\text{-}50}$ based on experimentally obtained secondary chemical shifts ($\Delta\delta^{13}$Ca-$\Delta\delta^{13}$Cb) shown in **(e)**. **f**) Experimentally obtained secondary chemical shifts of PEX19$_{1\text{-}50}$ bound to PEX38$_{65\text{-}134}$. **g)** TALOS-N Secondary structure propensity, **h)** experimentally obtained secondary chemical shifts and **i)** heteronuclear NOE experiments of free PEX38$_{65\text{-}134}$. **j)** TALOS-N. Secondary structure propensity, **k)** experimentally obtained secondary chemical shifts and **l)** heteronuclear NOE experiments of PEX38$_{65\text{-}134}$ bound to PEX19$_{1\text{-}50}$. In **g**) and **j**) propensities

for random coil, helix or beta strand secondary structure is shown in gray, blue and purple, respectively. Orange and yellow lines in h) – l) indicate differences in secondary structure and flexibility of free PEX38 (h, j) and PEX38 bound by PEX19.

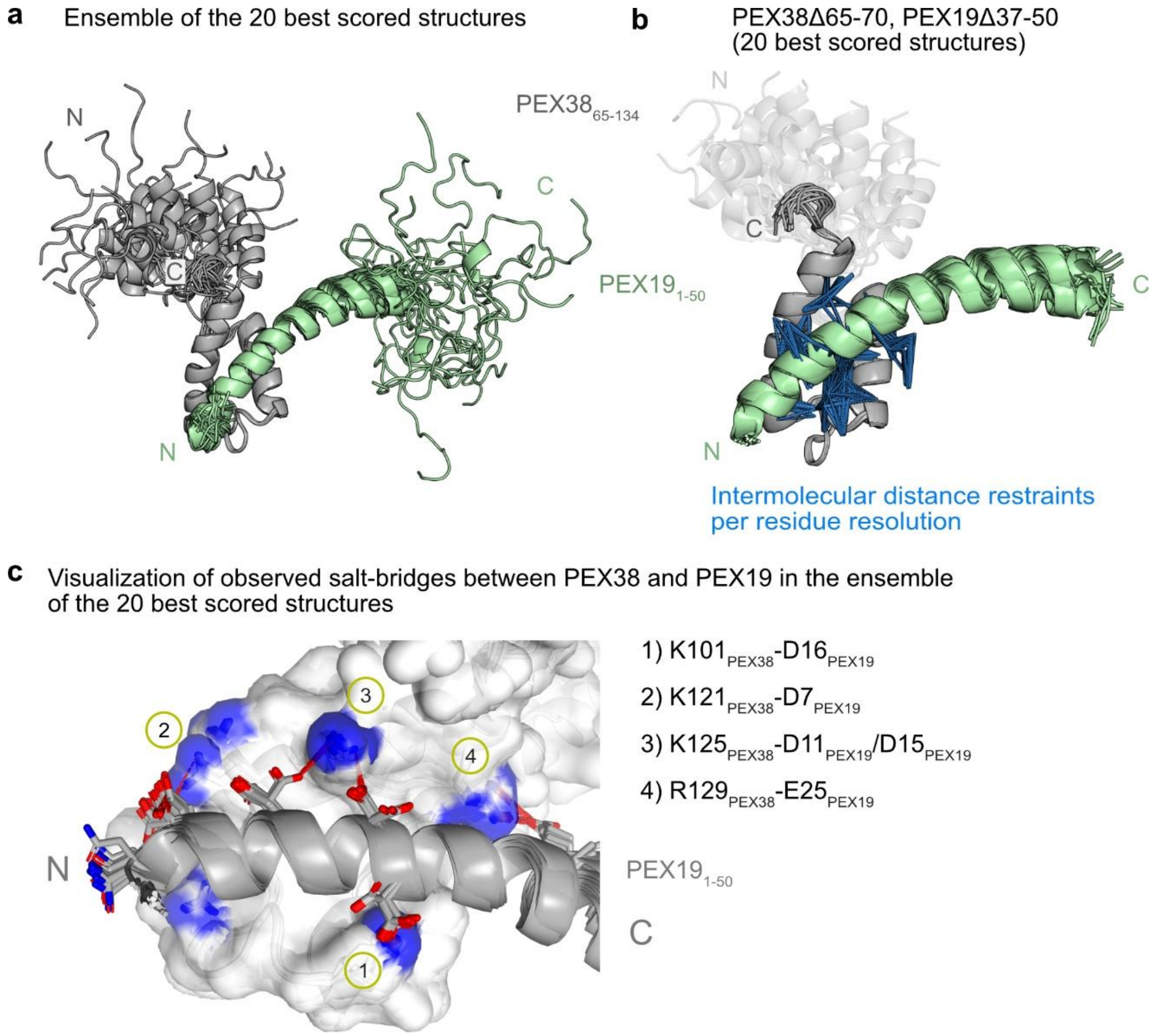

Fig. S7. Structure calculation of $PEX38_{65\text{-}134}$ in complex with $PEX19_{1\text{-}50}$. a) The 20 best scored structures of PEX38 (grey) and PEX19 (green) calculated with CYANA. **b)** Trimmed representation showing the only rigid structures (PEX38Δ65-70 and PEX19Δ37-50) with intermolecular with per residue resolution (not per atom; only one NOE per residue) visualized as blue dashed lines. **c)** Surface representation of PEX39 with cartoon representation of PEX19. Nitrogen atoms of Lys and Arg residues located in the PEX38 binding interface are colored in blue, while oxygen atoms of Asp and Glue residues (shown as sticks) of the PEX19 amphipathic helix are colored in red. Salt-bridges between PEX38 and PEX19 observed in the ensemble were visualized using PyMol 3.1.6.1 (Schrodinger) by detecting distances up to 4 Å between PEX38 Lys NZ or Arg NE and PEX19 Asp OD and Glu OE atoms and are indicated by red dashed lines. A hydrogen bond between PEX38 Arg118 and PEX19 Asn6 was not observed but would be possible as transient contact.

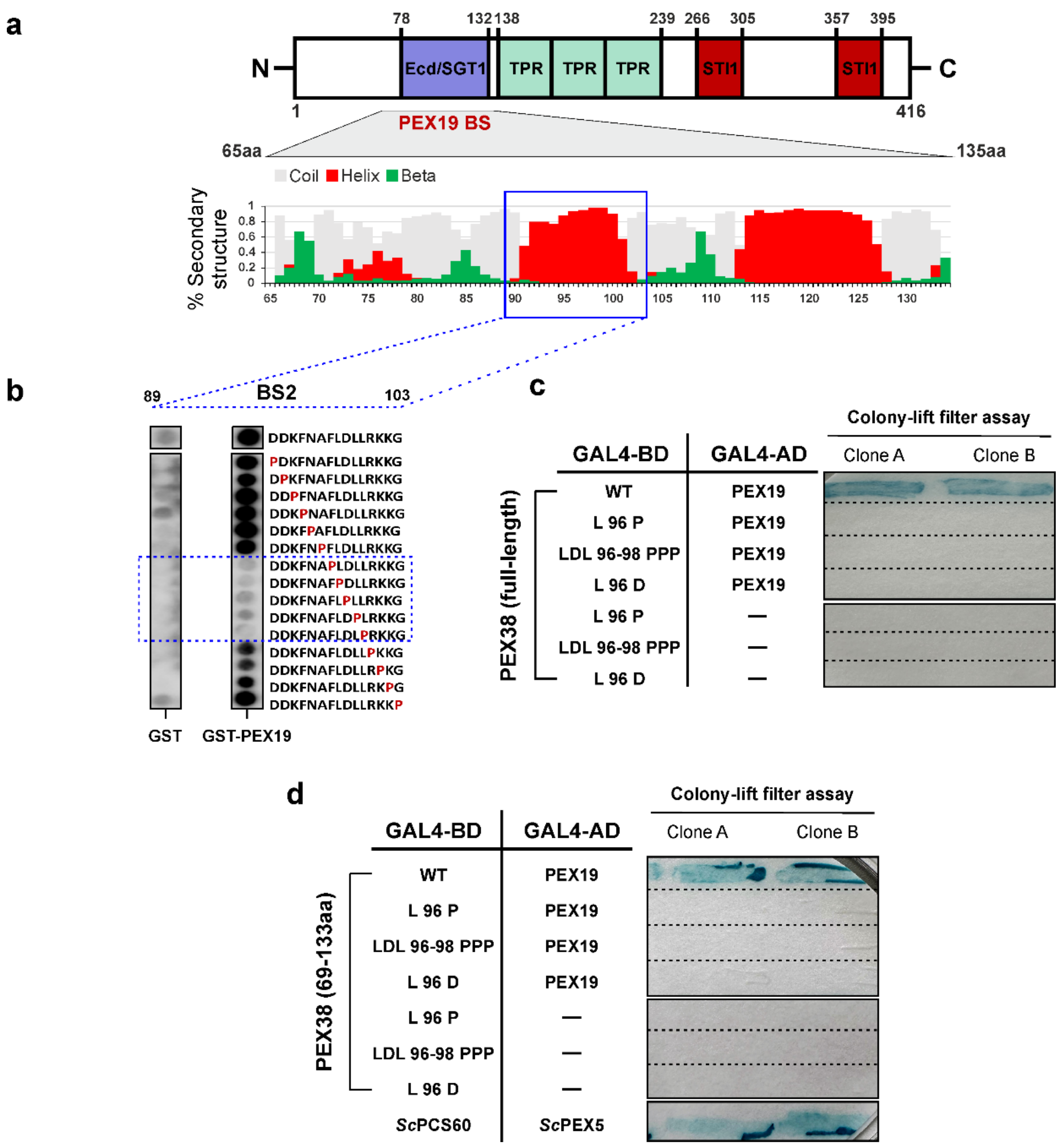

Fig. S8. Molecular characterization of the PEX19-PEX38 interaction. a) The schematic representation of PEX38 illustrates the domains predicted by the InterPro scan, with the PEX19 binding region highlighted in red. The experimentally determined secondary structure of the PEX19 binding domain (ECD/SGT1) of PEX38 is depicted as structural propensities obtained from secondary chemical shifts ($\Delta\delta^{13}$Ca-$\Delta\delta^{13}$Cb) and TALOS N (**Fig. S6g**). **b)** The identified PEX19 binding region in PEX38 predominantly comprises alpha-helical structures. From this region, the central PEX19 binding site, BS2, which consists of a 15-amino acid peptide, was selected for mutational analysis using a proline walk, as indicated by the blue dotted boxes. Synthetic 15-mer peptides with single proline substitutions at each position within the above-chosen region were tested for interaction with GST as a control or GST-PEX19. The wildtype peptide represents the unaltered sequence, while the proline walk indicates the proline substitution, shown in red font. Within the region spanning amino acids 89-103, replacement of the central residues FLDLL with proline, abolishes their interaction with PEX19, as denoted by the blue dotted box. **c, d)** Yeast two-hybrid (Y2H) assays were performed to investigate the effect of indicated mutations on the interaction between PEX38 and PEX19. Mutating the amino acid residues at positions L96 P/D and LDL 96-98 PPP abolished the interaction of both full-length PEX38 and its 69-133 fragment with PEX19. The assay was performed in triplicate using three different clones.

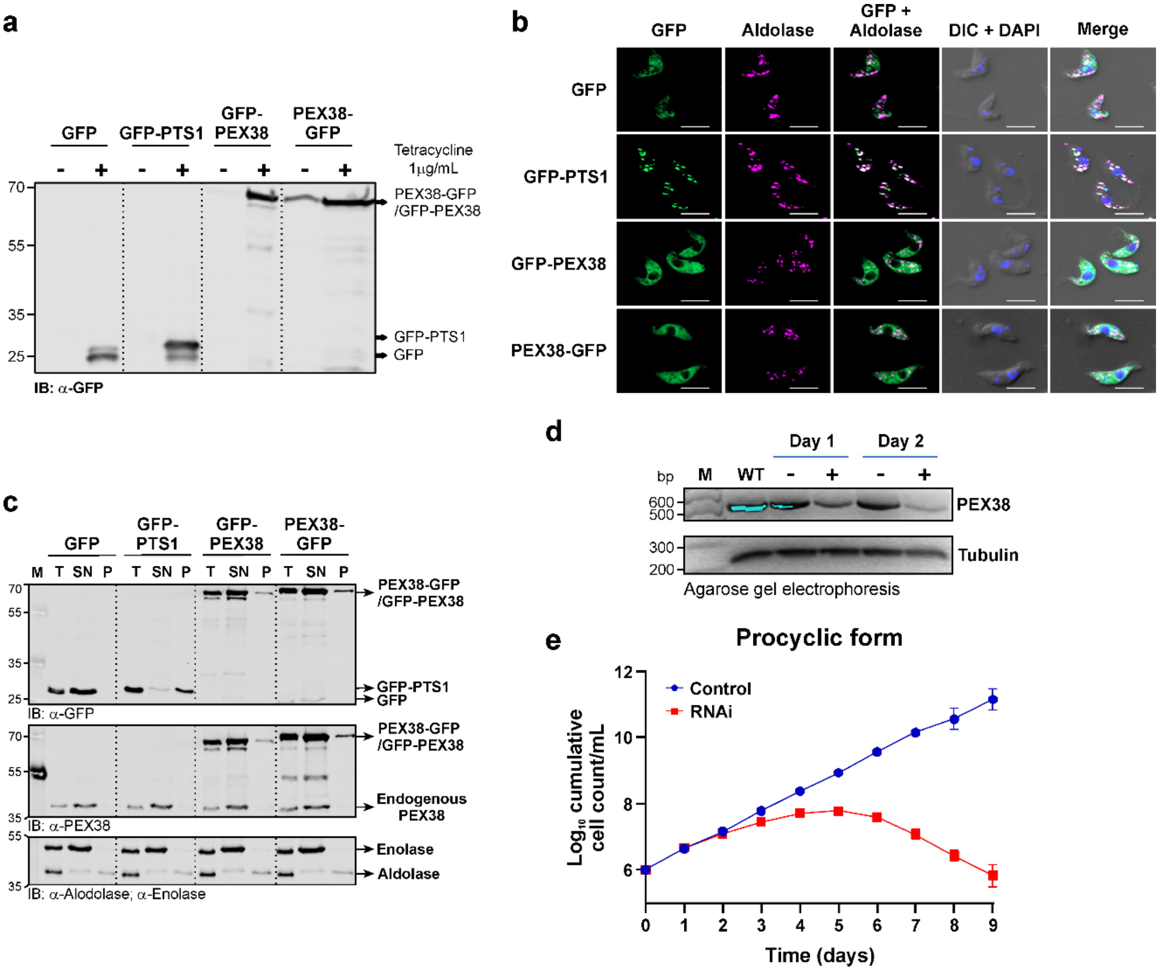

Fig. S9. Subcellular localization of GFP-tagged PEX38 proteins in PCF of *T. brucei* by fluorescence microscopy and cellular fractionation. a) Expression levels with and without tetracycline induction of GFP-fused constructs, including GFP, GFP-PTS1, and PEX38 tagged with GFP at the N- or C-terminus were analyzed by immunoblotting. **b)** Immunofluorescence microscopy of the localization of GFP fusion proteins as well as the glycosomal marker aldolase, and the DAPI-stained nucleus and kinetoplast in the same cell lines. The GFP-PTS1 constructs co-localized with the glycosomal marker aldolase (pseudocolored in magenta). In contrast, the positive control GFP exhibited a cytosolic pattern, as evident from the overall diffuse cell labelling. Similarly, the PEX38 constructs tagged with GFP at the N- or C-terminus also localized to the cytosol. Scale bar – 5 µm. **c)** Cellular fractionation was performed using the same cell lines mentioned above. Immunoblot analysis of fractions was performed using an anti-GFP antibody to detect indicated fusion proteins; enolase and GFP were monitored as cytosolic markers. The lanes represent the following samples: T, total lysate; SN, digitonin supernatant that contains cytosolic proteins; and P, digitonin pellet that contains organellar proteins. **d)** A semi-quantitative analysis of tubulin and PEX38 mRNA levels was performed using routine PCR with cDNA isolated from wild-type and RNAi bloodstream form cells, including uninduced samples and those with RNAi induction on days 1 and 2 (**Fig. 4b**). **e)** Growth curve demonstrating that PEX38 is an essential protein for the survival of *T. brucei* in the procyclic form.

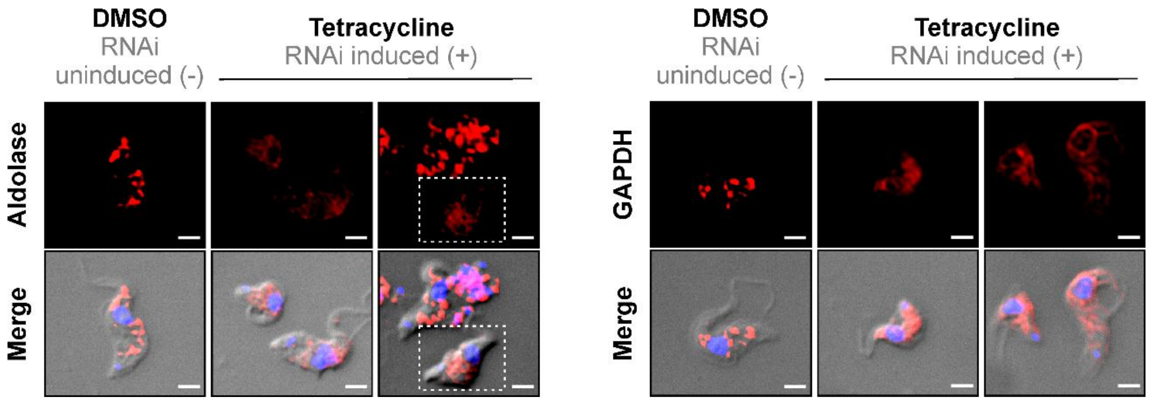

Fig. S10. Immunofluorescence microscopy of glycosomes upon PEX38 RNAi. On day 2 of PEX38 RNAi, both DMSO and tetracycline treated cells were analyzed for aldolase and GAPDH by immunofluorescence microscopy. In DMSO-treated cells, aldolase and GAPDH display a punctate glycosomal pattern. Upon PEX38 RNAi induction, glycosomal markers labelling was similar but puncta appeared less bright and more diffuse. Merge channel shows brightfield images with DNA stained by DAPI. The scale bar represents 2 μm.

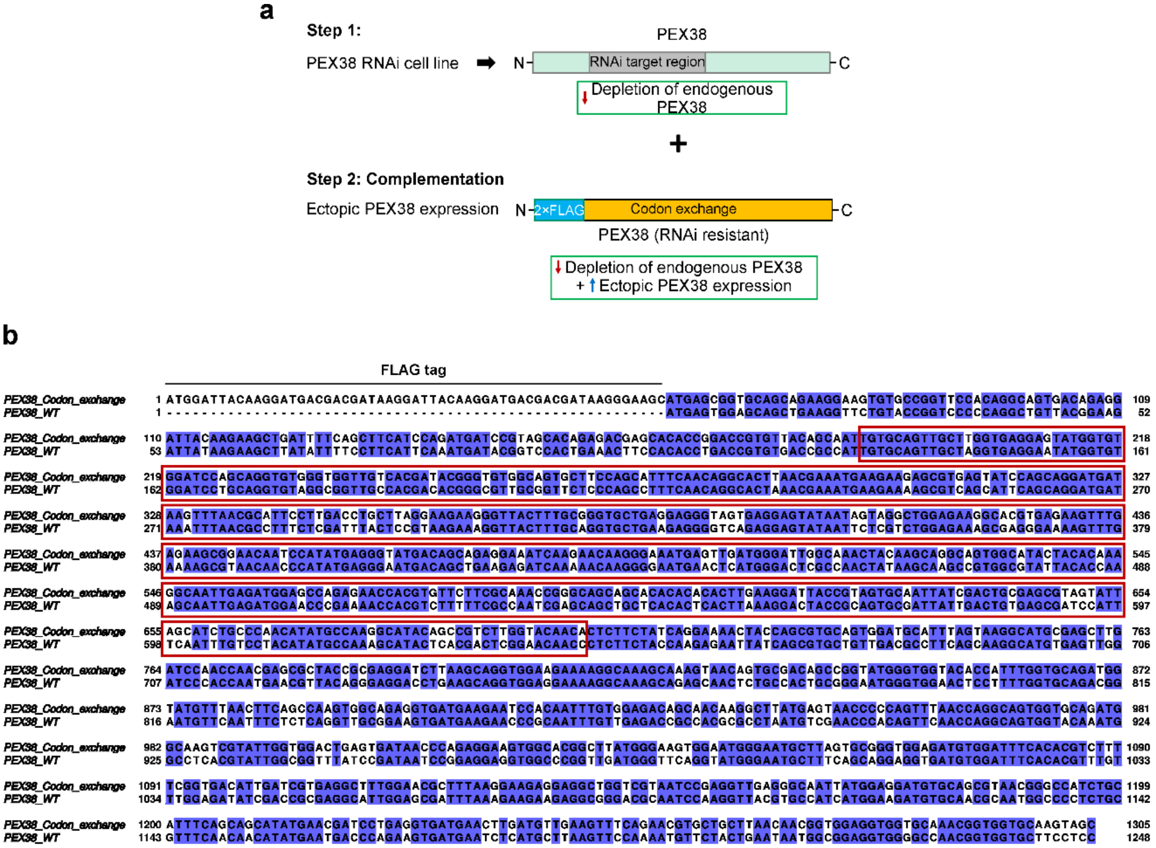

Fig. S11. a) Steps involved in generating an RNAi-resistant cell line for complementation assay. Step 1: Generation of a PEX38 RNAi cell line by genomic integration of a stem-loop RNAi construct containing the selection marker phleomycin through homologous recombination. This targets the coding region of PEX38 (marked in a grey box) resulting in the depletion of endogenous PEX38 (upper panel). Positive clones, verified by qRT-PCR for the efficient RNAi of PEX38, are further used in step 2 (lower panel). Step 2: Ectopic integration and expression of codon exchanged RNAi resistant 2×FLAG-tagged PEX38 for functional complementation (selection marker blasticidin). Both constructs are integrated into a tetracycline-inducible *Trypanosoma* cell line. Upon tetracycline induction, positive clones exhibit depletion of endogenous PEX38 and overexpression of 2×FLAG-PEX38 protein. **b)** Sequence alignment of wildtype PEX38 gene with a codon-exchanged synthetic gene encoding PEX38. The RNAi target region is marked by a red box. Multiple sequence alignment was performed using the Clustal Omega tool, and the aligned sequences were visualized using Jalview software with the percentage identity colour scheme.

a

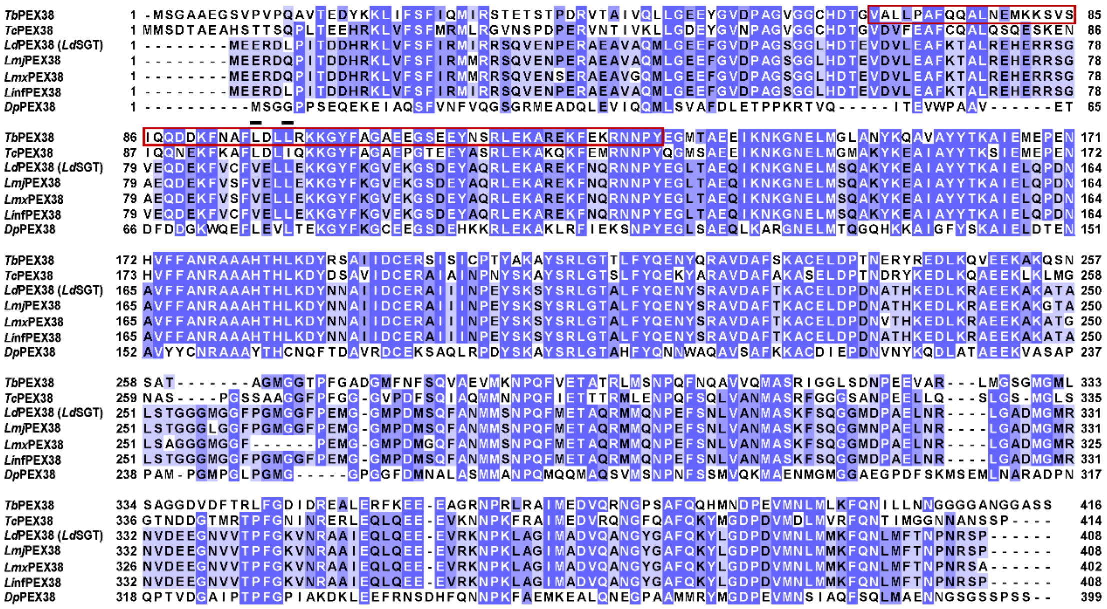

b

	*Tb*PEX38	*Tc*PEX38	*Ld*PEX38	*Lmj*PEX38	*Lmx*PEX38	*Linf*PEX38	*Dp*PEX38	
*Tb*PEX38		68.59	63.72	63.72	62.93	63.72	48.37	% Similarity
*Tc*PEX38	61.35		66.91	66.91	66.41	66.91	48.62	
*Ld*PEX38	56.12	59.55		98.28	97.26	100	52.63	
*Lmj*PEX38	56.37	60.04	98.28		97.51	98.28	51.87	
*Lmx*PEX38	55.47	59.7	97.26	97.51		97.26	52.62	
*Linf*PEX38	56.12	59.55	100	98.28	97.26		52.63	
*Dp*PEX38	35.08	36.84	39.84	39.59	39.09	39.84		
	% Identity							

c

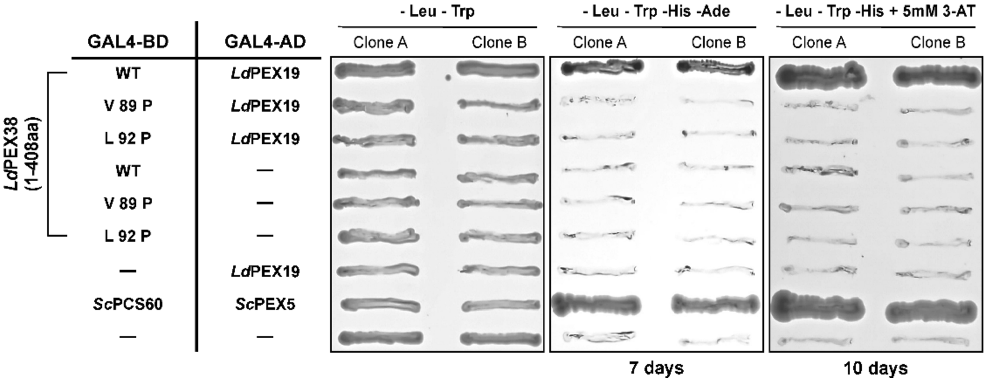

Fig. S12. Characterization of the PEX19-PEX38 interaction in clinically relevant *Leishmania* parasites. a) Multiple sequence alignment of the PEX38 protein sequences from the trypanosomatid parasites, including *Trypanosoma brucei*, *T. cruzi*, *Leishmania donovani*, *L. infantum*, *L. major*, *L. Mexicana* and *Diplonema papillatum*. Sequence conservation is color-coded based on percentage identity, with a conservation threshold of 30%. The red box highlights the identified PEX19 binding region within *T. brucei* PEX38, while the black lines indicate two conserved residues that are essential for interaction with PEX19. **b)** Analysis of percentage identity and similarity among PEX38 homologs across parasitic organisms using SIAS Homology Modelling. **c)** Yeast two-hybrid (Y2H) interaction analysis of *Ld*PEX38 (wild type or mutant; fused to GAL4 binding domain) and *Ld*PEX19 (fused to GAL4 activation domain) using colony-lift filter assays. The interaction between *Sc*PEX5 and *Sc*PCS60 served as a positive control. The assay was performed in triplicate using three different clones.

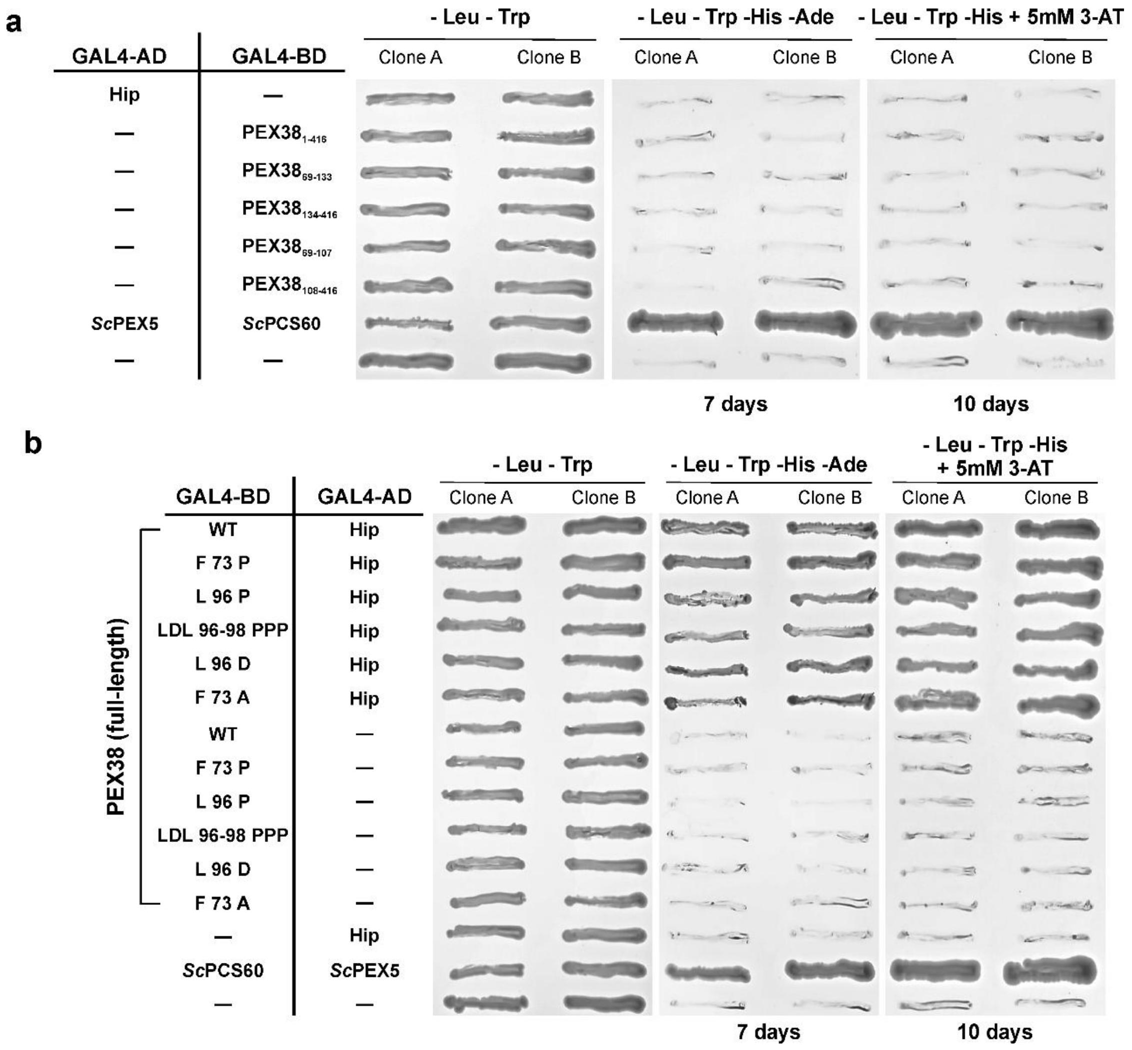

Fig. S13. a) A Y2H interaction assay was performed between full-length Hip and various PEX38 constructs fused to the GAL4-activation or -binding domain. The constructs were co-transformed into the PJ69-4A yeast strain and analyzed using a growth-based study. These results are negative controls for the study, as shown in **Fig. 5c**. **b)** The effect of PEX38 mutations that disrupt the interaction with PEX19 on the interaction with Hip protein was investigated using Y2H assay. Hip and PEX38 (wild type or mutants) constructs were fused to the GAL4-activation or -binding domain and co-transformed into the PJ69-4A yeast strain. The interactions were then analyzed using a growth-based assay.

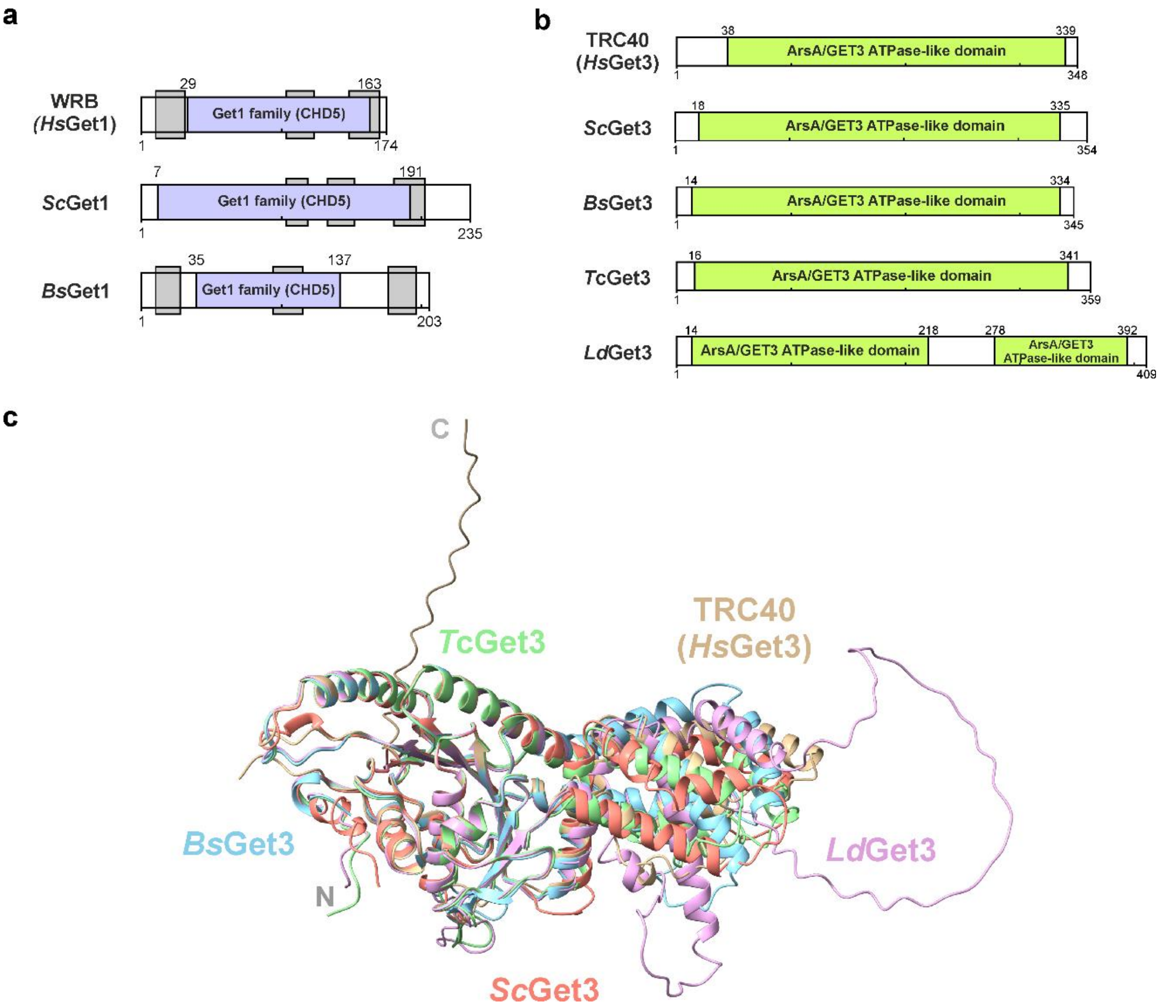

Fig. S14. Bioinformatic and structural analysis of Get1 and Get3 orthologs in kinetoplastids. **a)** Domain architecture of Get1 proteins from human *(Hs), S. cerevisiae (Sc),* and *Bodo saltans (Bs)*, revealed by InterPro domain searches. All proteins contain the conserved Get1 family domain (IPR028945), with grey boxes indicating predicted transmembrane segments. **b)** Domain analysis of Get3 homologs in kinetoplastids (*T. cruzi: Tc*, *L. donovani: Ld*, and *B. saltans: Bs*) alongside *Hs* and *Sc* Get3. All homologs contain the conserved ArsA/GET3 family domain (IPR025723) identified using the InterPro database. **c)** AlphaFold-predicted structures of kinetoplastid Get3 homologs (*Bs, Tc, Ld*) show high structural similarity to *Hs* and *Sc* Get3. Structural superpositions were performed using *Hs*Get3 as the reference. Colors indicate species: *Hs*Get3 (tan), *Sc*Get3 (salmon), *Bs*Get3 (blue), *Tc*Get3 (pale green), and *Ld*Get3 (plum).

Tables

Table S1. Isothermal titration calorimetry of PEX3865-134 titrated with PEX191-50 with n=3.

*Tb*PEX38 $_{65\text{-}134}$	K_D (μM)	∆H (kcal/mol)	∆G (kcal/mol)	-T∆S (kcal/mol)
*Tb*PEX19 $_{1\text{-}50}$	2.89 ± 0.15	-12.46 ± 0.24	-7.56 ± 0.03	4.93 ± 0.27

Table S2. Distance and angle restraints / violation statistics

**Statistic**	**Value**
**Distance and angle restraints**	
Total distance restraints	2752
**PEX19**	
intramolecular	783
intraresidual	209
sequential (|i-j|=1)	265
medium range (1<|i-j|<5)	308
long range (|i-j|>=5)	1
**PEX38**	
intramolecular	1745
intraresidual	416
sequential (|i-j|=1)	491
medium range (1<|i-j|<5)	563
long range (|i-j|>=5)	275
**Complex**	
intermolecular	224
long range (|i-j|>=5)	224
**Torsion angles (φ, ψ)[a] total**	166
PEX19 backbone	70
PEX38 backbone	96
**Violation and energy statistics[b]**	
Avg. distance viol. 0.1–0.2 Å	53.6 ± 18.4
Avg. distance viol. 0.2–0.3 Å	12.2 ± 4.6
Avg. distance viol. 0.3–0.4 Å	4.6 ± 2.1
>0.4 Å	3.5 ± 1.4
Maximal distance violation (Å)	0.85 ± 0.35
Avg. angle viol. <5°	14.2 ± 5.1
Avg. angle viol. >5°	4.6 ± 10.5
Maximal angle violation (°)	14.7 ± 7.3
Mean violation energy (total)	90.9 ± 31.1 kcal/mol
– Distance contribution	82.8 ± 28.6 kcal/mol
– Torsion contribution	8.1 ± 3.3 kcal/mol
Mean AMBER energy	–5147.3 ± 1715.8 kcal/mol
Bond length deviation	0.0034 ± 0.0011 Å
Bond angle deviation	1.165 ± 0.389 °

[a]. Justification for torsion angles, e.g. Molecule 1 TALOS+, Molecule 2 sugar pucker based on homonuclear TOCSY

[b]. Statistics computed for the deposited bundle of 20 violation energy best structure selected out of 30 amber energy best

Table S3. Structural quality (RMSD and Ramachandran)

**Statistic[b,c]**	**Value**
**RMSD**	
PEX19 backbone atoms	0.11 ± 0.05 Å
PEX19 heavy atoms	0.50 ± 0.17 Å
PEX38 backbone atoms	0.15 ± 0.05 Å
PEX38 heavy atoms	0.48 ± 0.09 Å
All molecules backbone atoms	0.17 ± 0.06 Å
All molecules heavy atoms	0.51 ± 0.08 Å
**Ramachandran[b,c,d]**	
Most favored regions	95.4 ± 1.2 %
Additionally allowed	4.6 ± 1.2 %
Generously allowed	0.0 %
Disallowed	0.0 %

[a]. Justification for torsion angles, e.g. Molecule 1 TALOS+, Molecule 2 sugar pucker based on homonuclear TOCSY
[b]. Statistics computed for the deposited bundle of 20 violation energy best structure selected out of 30 amber energy best
[c]. Based on structured residue range as defined by cyana command overlay: Molecule 1 (PEX19): 11-20, Chain ID: B (Sequence Range: 1-54); Molecule 2 (PEX38): 100-129, Chain ID: A (Sequence Range: 65-134)
[d]. Ramachandran plot, as defined by the program Procheck (2)

Table S4. Strains, Plasmids, and cloning strategy

No.	Expression in	Construct	Primer pair	Cloning strategy (Restriction sites)	Cloned in vector
1		GST-*Tb*PEX19$_{1-285}$ (full-length)	RE2926-RE7038		pGEX4T2
2		GST-*Tb*PEX19$_{31-285}$ (ΔN30)	RE7126-RE7038	BamHI/XhoI BamHI/XhoI	pGEX4T2
3		His$_6$-*Tb*PEX19$_{1-285}$ (full-length)	RE2926-RE7038		pET28a+
4		GST-*Tb*PEX38 (full-length)	RE7610-RE7611	EcoRI/XhoI	pGEX4T2
5	*E. coli*	*Tb*PEX19$_{1-285}$ (full-length) - His$_6$	RE8154-RE8135	NdeI/XhoI	pET21b
6		*Tb*PEX19$_{1-50}$- His$_6$	RE8341-RE8342	Quick change PCR	pET21b
7		GST-*Tb*PEX3 ΔN44	(3)		
8		His-SUMO-TbPEX19 1-50-GGY	TbPEX19 SG1+2/3+4 SLIM pETM13S		
9		His-SUMO-TbPEX38 65-134-GGY	TbPEX38 SG1+2/3+4 SLIM pETM13S		
10		GAL4 AD-*Tb*PEX19$_{1-285}$ (full-length)	(4)		
11		GAL4 BD-*Tb*PEX38$_{1-416}$ (full-length)	RE7508-RE7509		pPC97
12		GAL4 BD-*Tb*PEX38$_{69-107}$	RE8027-RE8028	SalI/NotI	pPC97
13		GAL4 BD-*Tb*PEX38$_{108-416}$	RE8031-RE7509		pPC97
14		GAL4 BD-*Tb*PEX38$_{69-133}$	RE8027-RE8029		pPC97
15	*S. cerevisiae*	GAL4 BD-*Tb*PEX38$_{134-416}$	RE8862-RE8863	Quick change PCR	pPC97
16		GAL4 AD-*Sc*PEX5$_{1-612}$	(5)		
17		GAL4 BD-*Sc*PCS60$_{1-543}$	(6)		
18		GAL4 BD-*Tb*PEX38$_{1-416}$ (F 73 P)	RE8866-RE8867	Quick change PCR	pPC97
19		GAL4 BD-*Tb*PEX38$_{1-416}$ (L 96 P)	RE8353-RE8354		pPC97

**20**		GAL4 BD-*Tb*PEX38$_{1-416}$ (LDL 96-98 PPP)	RE8235-RE8236		pPC97
**21**		GAL4 BD-*Tb*PEX38$_{1-416}$ (L 96 D)	RE8924-RE8925		pPC97
**22**		GAL4 BD-*Tb*PEX38$_{1-416}$ (F 73 A)	RE8922-RE8923		pPC97
**23**		GAL4 BD-*Tb*PEX38$_{69-133}$ (L 96 P)	RE8353-RE8354		pPC97
**24**		GAL4 BD-*Tb*PEX38$_{69-133}$ (LDL 96-98 PPP)	RE8235-RE8236		pPC97
**25**		GAL4 BD-*Tb*PEX38$_{69-133}$ (L 96 D)	RE8924-RE8925		pPC97
**26**		GAL4 BD-*Tb*PEX38$_{69-133}$ (F 73 A)	RE8928-RE8929		pPC97
**27**		GAL4 AD-*Tb*Hip$_{1-388}$	RE8112-RE8113	BglII/NotI	pPC97
**27**		GAL4 BD-*Tb*Hip$_{1-388}$	RE8112-RE8113		pPC86
**28**		GAL4 AD-*Tb*PEX19$_{31-285}$ (Δ30aa)	RE7092-RE7095	SalI/NotI	pPC86
**29**		GAL4 AD-*Sc*PEX19$_{1-342}$ (full-length)	RE9265-RE9266 (Insert)/ RE9267-RE9268 (Vector)	In-fusion cloning	pPC86
**30**		GAL4 AD-*Sc*PEX19$_{31-342}$ (Δ30aa)	RE9269-RE9270	Quick change PCR	pPC86
**31**		GAL4 AD-*Hs*PEX19$_{2-299}$ (full-length)	pAH07 (7)		pPC86
**32**		GAL4 AD-*Hs*PEX19$_{34-299}$ (Δ33aa)	RE9257-RE9258	Quick change PCR	pPC86
**33**		GAL4 BD-*Hs*SGTA$_{1-313}$	RE9251-RE9252	SalI/NotI	pPC97
**34**		GAL4 BD-*Sc*Sgt2$_{1-346}$	RE9249-RE9250	SalI/SacI	pPC97
**35**	***T. brucei***	*Tb*PEX38 RNAi (Fragment 1)	RE7528-RE7723	SpeI/NcoI	Ligate Fragments 1 and 2
**36**		*Tb*PEX38 RNAi (Fragment 2)	RE7529-RE7724	HindIII/NcoI	
**37**		*Tb*PEX38 RNAi (Stem-loop)	Ligated fragments 1 and 2	SpeI/HindIII	p2T7-177
**38**		2×FLAG-*Tb*PEX38; Codon exchanged	Gene synthesis	HindIII/ApaI	pHD1336

**39**		2×FLAG-*Tb*PEX38 (L 96 P); Codon exchanged	RE8858-RE8859	Quick change PCR	pHD1336
**40**		2×FLAG-*Tb*PEX38 (L 96 D); Codon exchanged	RE8868-RE8869		pHD1336
**41**		2×FLAG-*Tb*PEX38 (LDL 96-98 PPP); Codon exchanged	RE8860-RE8861		pHD1336
**42**		2×FLAG-*Tb*PEX38 (F 73 P); Codon exchanged	RE8864-RE8865		pHD1336
**43**		GFP-*Tb*PEX38 (full-length)	RE7573-RE7575	BstBI/ApaI	pGC1
**44**		*Tb*PEX38-GFP (full-length)	RE7573-RE7574		pGN1
**45**		GFP-SKL	(8)		

Table S5. Oligonucleotides

Primer	Sequence 5' to 3'
RE2926	GATCGGATCCATGTCTCATCCCGACAATGAC
RE7000	GCGCGAAATCGTCTGCGTTCAGG
RE7001	GCACGTACGGAGTCCATTGTACC
RE7038	CCGCTCGAGTTACACTGATGGTTGCACATCGGCAAGTCC
RE7092	AAGTGTCGACAAAGGCTCAGGAACGAGCGGCAACA
RE7095	AAGAGCGGCCGCCTACACTGATGGTTGCACATCGGC
RE7126	AATCGAGGATCCAAGGCTCAGGAACGAGCGGCAACA
RE7508	AAGGTCGACAATGAGTGGAGCAGCTGAAGGTTCTGT
RE7509	AAGACAGCGGCCGCTTAGGAGGAAGCACCACCGTTGGC
RE7528	AAACTAGTTGTGCAGTTGCTAGGTGAGG
RE7529	CCCAAGCTTTGTGCAGTTGCTAGGTGAGG
RE7573	AAAGTTCGAAGATGAGTGGAGCAGCTGAAGGTTCT
RE7574	AAGAGGGCCCGGGAGGAAGCACCACCGTTGGCC
RE7575	AAGAGGGCCCTTAGGAGGAAGCACCACCGTTGGCC
RE7610	AAGAGAATTCGAATGAGTGGAGCAGCTGAAGGTTC
RE7611	AAGAGCTCGAGTTAGGAGGAAGCACCACCGTTGGC
RE7723	AAGAACCATGGACATGCCTTGCTGAAGGCGTCAAC
RE7724	AAGACCCATGGGGTTGTTCCGAGTCGTGAGTATG
RE8027	AGACGTCGACAGTTCTCCCAGCCTTTCAACAGG
RE8028	AGACGCGGCCGCTTAACCTGCAAAGTAACCTTTCT
RE8029	AGACGCGGCCGCTTAATATGGGTTGTTACGCTTTT
RE8031	AAGACGTCGACAGCTGAAGAGGGGTCAGAGGAGTA
RE8112	GGAGATCTCGATGCATACTCTGTCCTCATGTGAT
RE8113	AGAAAGCGGCCGCTTAATCCAACTCGTCAGGGTC
RE8135	AACCGCTCGAGTCCGCTCACTGATGGTTGCACATCGGCAAGTCC
RE8154	GAACAAGGCATATGTCTCATCCCGACAATGACGCC
RE8235	CTTTCCACCGCCTCTCCGTAAGAAAGGTTACTTTGC
RE8236	AGAGGCGGTGGAAAGGCGTTAAATTTATCATCCTGC
RE8341	CAGAGTTGAGCGGACTCGAGCACCAC
RE8342	GTCCGCTCAACTCTGCAGTTGCACTCTTTTG
RE8353	CGCCTTTCCCGATTTACTCCGTAAGAAAGGTTAC
RE8353	CGCCTTTCCCGATTTACTCCGTAAGAAAGGTTAC
RE8354	AAATCGGGAAAGGCGTTAAATTTATCATCCTG
RE8354	AAATCGGGAAAGGCGTTAAATTTATCATCCTG
RE8858	CGCATTCCCTGACCTGCTTAGGAAGAAGGGTTAC
RE8859	AGGTCAGGGAATGCGTTAAACTTATCATCCTG

RE8860	ATTCCCTCCCCCGCTTAGGAAGAAGGGTTACTTTGCGG
RE8861	AGCGGGGGAGGGAATGCGTTAAACTTATCATCCTGC
RE8862	GGTCGACAGAGGGAATGACAGCTGAAGAGA
RE8863	TTCCCTCTGTCGACCTCGACGATACAGTCA
RE8864	TCCAGCACCTCAACAGGCACTTAACGAAATGAAGA
RE8865	TGTTGAGGTGCTGGAAGCACTGCCAC
RE8866	CCCAGCCCCTCAACAGGCACTAAACGAAATGAAG
RE8867	TGTTGAGGGGCTGGGAGAACCGCAAC
RE8868	CGCATTCGATGACCTGCTTAGGAAGAAGGG
RE8869	AGGTCATCGAATGCGTTAAACTTATCATCCTGC
RE8922	CCCAGCCGCTCAACAGGCACTAAACGAAATG
RE8923	TGTTGAGCGGCTGGGAGAACCGCAAC
RE8924	CGCCTTTGACGATTTACTCCGTAAGAAAGGTTAC
RE8925	AAATCGTCAAAGGCGTTAAATTTATCATCCTGC
RE8928	CCCAGCCGCTCAACAGGCACTAAACGAAATGAAG
RE8929	TGTTGAGCGGCTGGGAGAACTGTCGACC
RE9249	AGGACGTCGACGATGTCAGCATCAAAAGAAGAA
RE9250	AAGACGAGCTCCTATTGCTTGTTCTCATTGTCTGG
RE9251	AAGACGTCGACGATGGACAACAAGAAGCGC
RE9252	AAGACGGCGGCCGCTCACTCCTGCTGGTCGTCGTT
RE9257	GGTCGACCCCCTCCCCAGCACCCCCT
RE9258	GGGAGGGGGTCGACCCACCCTCTTTTT
RE9265	AAAGAGGGTGGGTCGACCATGAATGAAAACGAGTACGATAATTTTGAT GAT
RE9266	GGCCGCACTAGTAGATCTTTATTGTTGTTTGCAACCGTCGGTTAATT
RE9267	AGATCTACTAGTGCGGCCGC
RE9268	GGTCGACCCACCCTCTTTTTTTG
RE9269	GGTCGACCGTGCAAGCGAAGGGTTCTGTG
RE9270	CTTGCACGGTCGACCCACCCTCTTT
TbPEX19_SG1	ATGTCTCATCCCGACAATGACG
TbPEX19_SG2	TTTCGGGCTTTGTTAGTATCCTCCGCTCAACTCTGCAGTTGCACTC
TbPEX19_SG3	GAACAGATTGGTGGCATGTCTCATCCCGACAATGACG
TbPEX19_SG4	GCTCAACTCTGCAGTTGCACTC
TbPEX38_SG1	ACGGGCGTTGCGGTTC
TbPEX38_SG2	TTTCGGGCTTTGTTACTCATATGGGTTGTTACGCTTTTCAAAC
TbPEX38_SG3	GAACAGATTGGTGGCACGGGCGTTGCGGTTC
TbPEX38_SG4	CTCATATGGGTTGTTACGCTTTTCAAAC

Dataset S1 (separate file). Mass spectrometry proteomics data related to Figures 1 and S2. Dataset S1, Tab 1 presents quantitative results. Dataset S1, Tab 2 lists analysis parameters.

Extended Methods

Cloning

The expression plasmid constructs and cloning strategies for *Escherichia coli*, yeast, and *Trypanosoma* are detailed in **Table S4**, while the oligonucleotide sequences are provided in **Table S5**. The Strep-Hip construct was cloned using the FastCloning method, as described previously (9). Overlap extension PCR was used to generate the point mutations and gene fragment deletions (PEX38 134-416). Automated Sanger sequencing was employed to verify the sequences of all constructs, mutations, and gene fragment deletions.

For structural characterization, cloning of PEX38 (65-134) and PEX19 (1-50-SGGY) into a pET SUMO expression vector was performed using site-directed ligase-independent mutagenesis (SLIM). To this end, an extended version was applied to implement inserts using the same fashion of short and tail primers. The vector backbone and inserts were amplified by polymerase chain reaction (PCR) amplification using the according short and tail primers (**Table S5**) to generate overlaps with sticky ends. The backbone amplificants and inserts were mixed with a 5-fold molar excess of insert and annealed during the SLIM cycle. The annealed vector was directly transformed into DH10b cells for DNA amplification.

For PEX38 RNA interference, a stem-loop construct was generated using two fragments of the PEX38 gene's RNAi target region. Fragment 2 (HindIII/MfeI) contained an additional 50-60 base pairs compared to Fragment 1 (SpeI/MfeI), resulting in a step-loop structure. Fragments 1 and 2 were amplified by PCR using primers designed for the RNAi target region using a web-based tool (10), as specified in **Table S4**. Subsequently, both fragments were subjected to digestion with the common enzyme MfeI, followed by ligation with 250 ng of each fragment. After reaction cleanup, the ligated fragment was further digested with SpeI and HindIII and then cloned into the p2T7-177 vector digested with SpeI-HindIII. For the *in cellulo* complementation assay, a codon-exchanged, RNAi-resistant PEX38 gene with an N-terminal 2×-FLAG tag was custom-synthesized and obtained from GeneCust, France.

Protein expression and purification

E. coli strain TOP10 was used for all plasmid amplifications, with liquid cultures grown at 37°C under continuous shaking in LB medium containing either 100 µg/mL Ampicillin or 50 µg/mL kanamycin. The BL21 *E. coli* strain was used for heterologous expression of recombinant GST, GST-PEX19 full-length ($PEX19_{FL}$) or GST-PEX19 lacking N-terminal 30 amino acids ($PEX19_{\Delta N30}$), GST-PEX38 (full-length or 69-133), GST-PEX3Δ44, PEX19-His, PEX19 N50-His, and StrepII-Hip fusion proteins. The expression plasmids encoding these proteins were transformed into the BL21 *E. coli* strain. LB medium, containing ampicillin, was inoculated with single colonies and incubated overnight at 37°C while shaking. The following day, the cultures were reinoculated at 0.1 OD_{600}/mL and further incubated at 37°C with shaking until cell density reached 0.6 OD_{600}/mL. Protein expression was induced with 1 mM IPTG for 4 h at 30°C, except for GST-PEX3Δ44, which was induced with 0.4 mM IPTG for 16 h at 16°C. Harvested cell pellets were stored at −20°C before use.

For protein purification, harvested cell pellets were resuspended in PBS with protease inhibitors (5 µg/mL Antipain, 2 µg/mL Aprotinin, 0.35 µg/mL Bestatin, 6 µg/mL Chymostatin, 2.5 µg/mL Leupeptin, 1 µg/mL Pepstatin, 0.1 mM PMSF, 25 µg/mL DNAse, and 1 mM DTT). Cells were disrupted using EmulsiFlex, and unbroken cells were removed by centrifugation at 4,000g (rotor SX4400, Beckman Coulter) for 15 min. The resulting supernatant (SN1) was subjected to high-speed centrifugation at 24,000 g for 1 h (rotor SS-34, Thermo Scientific), yielding supernatant 2

(SN2), a soluble fraction that included overexpressed proteins. Proteins were purified by affinity chromatography using Glutathione Agarose 4B beads for GST-tagged proteins, Protino Ni-NTA Agarose for His-tagged proteins, and Strep-Tactin®XT 4Flow® resin for Strep-tagged proteins. SN2 was incubated with the pre-equilibrated resin for the respective tagged proteins for 2 h in a tube rotator. After collecting the flow-through using a gravity flow column, the protein-bound beads were washed five times with PBS. Proteins were eluted with the appropriate elution buffer: 10 mM reduced glutathione in 50 mM Tris-Cl (pH 8.0) for GST-tagged proteins and 200 mM imidazole in PBS (pH 8.0) for His-tagged proteins. Purification of Strep-tagged proteins was performed according to the manufacturer's protocol (Iba, cat. no. 2-5010-002). The buffer of the eluted proteins was exchanged to PBS using Amicon centrifugation tubes with a molecular weight cut-off (MWCO) of 10 kDa. The concentration of the proteins was determined by the Bradford method (Thermo, Coomassie Plus assay kit), and protein aliquots were stored at −80°C. All purification steps were performed at 4°C. PBS: 10 mM Na_2HPO_4. $2H_2O$, 1.76 mM KH_2PO_4 137 mM NaCl, 2.7 mM KCl, pH 7.4

Trypanosoma cell culture and transfection

In this study, *T. b. brucei* bloodstream form 90-13 (BSF) and procyclic form 29-13 (PCF) cell lines, which co-express T7 RNAP and TetR, were used. PCF cells were grown in SDM-79 medium at 28°C (11) and the BSF cells were cultured in HMI-11 medium at 37°C with 5% CO_2 (12). Both cell lines were supplemented with heat inactivated 10% fetal bovine serum. The PCF cultures were maintained at a density of 1 × 10^6 to 30 × 10^6 cells/mL, while the BSF cultures were kept in the logarithmic growth phase (cell density below 2×10^6 cells/mL). Plasmid constructs linearized with the NotI restriction enzyme were transfected into PCF cells as described in (13) or BSF cells as described in (14), to integrate stably into the ribosomal RNA locus and the resulting clones were selected using the below described antibiotics. for PCF clones: 10 µg/ml blasticidin for pGN1/pGC1, or 5 µg/ml phleomycin for p2T7-177. For BSF clones: 5 µg/ml blasticidin for pGN1/pGC1, or 2.5 µg/ml phleomycin for p2T7-177.

Affinity isolation of PEX19 interactome using in vitro pulldown

The PCF cells were grown to a density of 20 × 10^6 cells/mL, harvested (∼500 mL), and snap frozen. Frozen cells were thawed, resuspended in PBS containing 1x protease inhibitor cocktail (PIC) and 1 mM EDTA, and permeabilized with 0.08 mg of digitonin/mg of protein. After a 5-min incubation at room temperature, the cell suspensions were centrifuged at 24,000 g (rotor SS-34, Thermo Scientific) for 30 min to obtain the cytosol-enriched fraction. The sedimented organellar pellet was then treated with a higher digitonin concentration of 3 mg/mg of protein and incubated for 30 min at 4°C on a Mini Rocker. Finally, the samples were centrifuged at 24,000g (rotor SS-34, Thermo Scientific) for 30 min to yield the solubilized organelle-enriched fractions.

Concurrently, 350 µg of recombinant proteins, including GST-PEX19FL, GST-PEX19 ΔN30, and GST as a negative control, were allowed to bind to 100 µL of settled glutathione agarose beads by gently rotating the tubes at 4°C for 2 h. After incubation, the beads were washed with PBS to remove unbound proteins. The *Trypanosoma* cytosol or organelle enriched fractions, prepared using the digitonin treatment, were then added to the glutathione agarose beads pre-bound with the recombinant proteins in separate tubes as follows: 1. GST alone, 2. GST + Cytosol fraction, 3. GST + organelle fraction, 4. GST-$PEX19_{FL}$ alone, 5. GST-$PEX19_{FL}$ + Cytosol fraction, 6. GST-$PEX19_{FL}$ + organelle fraction, 7. GST-$PEX19_{\Delta N30}$ alone, 8. GST-$PEX19_{\Delta N30}$ + Cytosol fraction, 9. GST-$PEX19_{\Delta N30}$ + organelle fraction. The tubes were then gently rotated at 4°C for 2 h to facilitate the binding of *Trypanosoma* proteins to GST-$PEX19_{FL}$ and GST-$PEX19_{\Delta N30}$. Prior to elution, the beads were washed five times with PBS, with each wash performed by centrifugation

to remove unbound proteins. Specifically bound proteins were eluted using 2 units of thrombin in 120 µL PBS, with incubation at 16°C for 16 h to ensure efficient thrombin cleavage. Finally, the eluted samples were analyzed by SDS-PAGE analysis followed by silver staining (described in (15)) and subjected to proteomic analysis to identify the bound proteins.

Proteolytic in-gel digestion

Thrombin-eluted complexes prepared as described above in triplicates were processed for tryptic in-gel digestion as described previously (16). Approximately 35 µL of each eluate was separated on 14% Tris-glycine Novex Wedgewell denaturing gels (Invitrogen) and visualized using colloidal Coomassie Brilliant Blue. Each gel lane was cut into 10 slices. Following destaining, gel slices were treated with 5 mM Tris(2-carboxy-ethyl) phosphine prepared in 10 mM ammonium bicarbonate (ABC) to reduce cysteine residues (30 min at 37 °C). Free thiol groups were then alkylated with 50 mM chloroacetamide/10 mM ABC (30 min at room temperature). In-gel digestion was carried out overnight at 37 °C using 0.06 µg of sequencing-grade trypsin (Promega) dissolved in 10 mM ABC per gel slice. Resulting peptides were extracted by two successive rounds of incubation in 0.05% (v/v) trifluoroacetic acid (TFA) and 50% (v/v) acetonitrile (ACN) using an ultrasonic bath (10 min at 4 °C each). Corresponding peptide-containing supernatants were pooled, dried under vacuum, and subsequently desalted with StageTips[48]. To this end, StageTips were conditioned sequentially with methanol, 80% ACN in 0.5% acetic acid (v/v), and 0.5% (v/v) acetic acid. Peptides were loaded onto StageTips, washed twice with 0.5% (v/v) acetic acid, and eluted with 80% ACN/0.5% acetic acid (v/v). Peptides were dried and stored at −80 °C until further analysis.

Liquid chromatography-mass spectrometry analysis

Dried peptide mixtures were resuspended in 0.1% TFA and analyzed by nano high-performance liquid chromatography-electrospray ionization-tandem mass spectrometry (Nano-HPLC-ESI-MS/MS) using an Orbitrap Elite hybrid mass spectrometer (Thermo Fisher Scientific, Bremen, Germany) coupled to an UltiMate 3000 RSLCnano HPLC system (Thermo Fisher Scientific, Dreieich, Germany). The RSLC system was operated with C18 pre-columns (nanoEase M/Z Symmetry C18; 20 mm length, 0.18 mm inner diameter) and an analytical C18 reversed-phase nano LC column (nanoEase M/Z HSS C18 T3; 250 mm length, 75 mm inner diameter, 1.8 mm particle size, 100 Å packing density). A binary solvent system was employed for peptide separation, composed of 4% (v/v) dimethyl sulfoxide (DMSO)/0.1% (v/v) formic acid (FA) (solvent A) and 30% (v/v) ACN/48% (v/v) methanol/4% (v/v) DMSO/0.1% (v/v) FA (solvent B). Peptides equivalent to 1 µg of protein were loaded, pre-concentrated and washed on the precolumn for 5 min using solvent A and a flow rate of 10 µL/min. Peptides were eluted using the following gradient: 1–7% solvent B in 5 minutes, 7–65% B in 30 min, 65-80% B in 15 min, and 3 min at 80% B at a flow rate of 300 ml/min. Eluted peptides were directed to a fused silica emitter for electrospray ionization using a Nanospray Flex ion source with a DirectJunction adaptor, applying a spray voltage of 1.8 kV and a capillary temperature of 200°C. Mass spectrometric data were acquired in data-dependent mode using the following parameters: MS precursor scans at *m/z* 370–1700 with a resolution of 120,000 (at *m/z* 400); automatic gain control (AGC) of 1×10^6 ions; a maximum injection time (IT) of 200 ms; a TOP20 method for low-energy collision-induced dissociation of multiply charged precursor ions with a normalized collision energy of 35%, an activation q of 0.25, and an activation time of 10 ms; AGC for MS/MS scans of 5×10^3 ions with a maximum IT of 150 ms; and a dynamic exclusion time of 45 sec.

MS data analysis

For the analysis of mass spectrometric data, the MaxQuant software package (version 1.6.10.43) (17) and its integrated search engine Andromeda (18) were employed. Raw data were searched against all protein sequences of *T. brucei* (strain Lister 427, TREU427) obtained from the TriTrypDB (release 48; https://tritrypdb.org/tritrypdb/app) to receive information about proteins present in the sample. 'Trypsin/P' was selected as proteolytic enzyme, allowing up to three missed cleavages, and mass tolerances were set to 20 ppm for precursor ions and 0.5 Da for fragment ions. The options 'match between runs' and 'iBAQ' (i.e., intensity-based absolute quantitation) were activated. Protein identification was based on the detection of at least one unique peptide of seven or more amino acids, applying a false discovery rate of 0.01 to both peptide and protein identifications. For all other parameters, MaxQuant default settings were used, including the fixed modification of carbamidomethylation for cysteine residues and variable modifications set for N-terminal acetylation and methionine oxidation.

For data analysis and visualization, MaxQuant results were processed using the autoprot Python module (v0.2) (19). To identify proteins specifically associated with PEX19FL or PEX19ΔN30, PEX19FL/GST, PEX19ΔN30/GST and PEX19ΔN30/PEX19FL protein abundance ratios were calculated based on iBAQ MS intensities, for both cytosolic and organellar fractions. Proteins were required to be identified in at least two out of three replicates per experimental condition. First, iBAQ values were $\log_2$ transformed. Missing values (i.e., in case a protein was only identified in 2/3 replicates) were imputed by drawing random values from a distribution matching the iBAQ value distribution of the existing values shifted downward by 1.3 standard deviations and scaled to a width of 3%. Log_2 abundance ratios as indicated above were calculated and normalized between equal conditions using cyclic loess normalization (20). The rank-sum test implemented in autoprot based on the R package RankProd (version 3.11) (21) as then used to determine proteins specifically enriched with a given bait protein.See **Dataset S1** for detailed results. A Jupyter notebook providing documentation of the analysis pipeline and statistical tools used is available at https://github.com/ag-warscheid/Tb_PEX38_manuscript.

Yeast two-hybrid analysis (Y2H)

Yeast two-hybrid assays, including both a colony-lift filter assay (also referred to as a plate-based assay) and a growth-based assay, were performed to investigate protein-protein interactions. As described in (4), the colony-lift filter assay was conducted using the *S. cerevisiae* wild-type strain PCY2. The growth-based Y2H assay, as described in (22), was performed using the *S. cerevisiae* strain PJ69-4A. Yeast cells were transformed using the traditional lithium-acetate method, and the interaction analysis was carried out for both assays as described in (4) and (22). The interaction between the GAL4 activation domain fused to *Sc*PEX5 and the GAL4 DNA-binding domain fused to *Sc*PCS60 was used as a positive control for the study. Additionally, the GAL4-AD fusion of PEX19, Hip, or *Ld*PEX19, as well as the various GAL4-BD fusions of PEX38 (wild-type or mutants), Hip, or *Ld*PEX38 (wild-type or mutants), were tested for autoactivation, serving as negative controls.

Peptide array

To identify the binding sites and conduct mutational analysis, *Tb*PEX19 and *Tb*PEX38 peptide arrays were obtained. The immobilized peptides comprised 25 amino acids for PEX19 and 15 amino acids for PEX38, sequentially overlapping by 20 and 13 residues, respectively, to represent the entire sequences of PEX19 and PEX3. These peptides were synthesized on a cellulose membrane, as described previously in (23). For the proline walk, 15-amino-acid-long peptides, i.e.,

PEX38 89-103, were used, substituting each residue with a proline. The peptide array was first activated with ethanol for 10 min with gentle shaking, followed by three 10-min washes with TBS. Subsequently, the peptide array was incubated with a blocking buffer (5% fat free milk powder + 0.05% tween 20 + 5% Sucrose in TBS) for 1 h at room temperature. The purified recombinant proteins GST-PEX19, GST-PEX38, or GST alone with a final 1 μM (15 mL) concentration were incubated with the protein arrays for 1 hour at 4°C. Subsequently, the arrays were subjected to three 10-min washes in a TBS buffer at room temperature. This was followed by a ~16 h incubation with an anti-GST (Sigma, 1:2,000 in blocking buffer) monoclonal antibody at 4°C. After three more TBS washes, a secondary antibody (Horseradish peroxidase-coupled anti-mouse IgGs, 1:5,000 in blocking buffer) was applied, and the arrays were incubated for 1 h at room temperature. Finally, the arrays were scanned using a chemiluminescence substrate (WesternBright Sirius) and the Azure Sapphire biomolecular imager. TBS: 50 mM Tris, 137 mM NaCl, 2.7 mM KCl, pH 8.

In vitro pull-down assay of PEX19 N50-His_6

For the in vitro pull-down assay, 10 μL of settled glutathione agarose beads were separately incubated with 100 μg of recombinant GST and GST-PEX38 proteins. The incubation was carried out for 2 h at 4°C with gentle rotation. After washing the beads three times with TBS to remove unbound proteins, 20 μg of recombinant PEX19 N50-His_6 containing 5% sucrose was added to the beads. This allowed the PEX19 protein to bind to either the GST-PEX38 or the control GST for an additional 2 h at 4°C with gentle rotation. Following three more TBS washes, the bound proteins were eluted with 40 μL of 10 mM reduced glutathione in 50 mM Tris, pH 8. The eluted samples were analysed using SDS-PAGE followed by Coomassie staining and immunoblotting.

Cleavage of GST-tag with biotinylated thrombin

To obtain tag-free proteins for the displacement/competition experiments, 2.5 mg of GST-PEX38 (full-length) was incubated with 1.5 units of biotinylated thrombin (69672-50UN, Sigma-Aldrich) in 1 mL of PBS at 4°C for ~16 h. The biotinylated thrombin was then captured using a 50 μL magnetic slurry of Dynabeads™ MyOne™ Streptavidin T1 (65601, Invitrogen). After a 60-min incubation at 4°C with gentle rotation, the samples in the microfuge tube were magnetically separated by placing the tube in a magnet rack for 2-3 min. The tube containing a mixture of the cleaved GST tag and the tag-free protein was carefully decanted. To remove the cleaved GST tag from the tag-free proteins of interest, 350 μL of settled glutathione agarose beads were added and incubated at 4°C for 2 h. Following the incubation, the flow-through containing the tag-free protein was collected. The beads were either denatured or eluted with 10 mM reduced glutathione in 50 mM Tris. The samples collected from each step were analyzed using SDS-PAGE and Coomassie staining to assess the cleavage efficiency and protein purity. In addition, the same tag-free PEX38 65-134 that was used for the structural study was also used for the competition assay.

AlphaScreen-based assay

The AlphaScreen-based assay was employed for the binding study, competition, or displacement assay, as well as to assess the formation of the ternary complex. To investigate the interaction between the C-terminal His6-tagged PEX19 (full-length and N50) and N-terminal GST-tagged PEX38 (full-length and 69-133), the proteins were used at a concentration of 30 nM in the AlphaScreen system. The protein solution was prepared in PBS (pH 7.4) with 0.5% BSA, and the donor and acceptor beads were prepared in the same buffer with 0.05% Tween 80, both at a concentration of 5 μg/mL. The bead information and assay details were as described previously (4). The binding assay was performed in three biological replicates, with 6 technical replicates each.

The competition assay was designed to demonstrate that untagged PEX38 (full-length or 65-134) can displace GST-tagged PEX3Δ44 from binding to PEX19-His. Here, 30 nM of PEX19-His was added to a 384-well plate, followed by the addition of 30 nM of GST-PEX3Δ44. Serially diluted untagged proteins, either full-length PEX38 or the 65-134 amino acid region of PEX38, were then added. The dilution was a 2-fold series with 12 points, starting from 0 to 3660 nM for full-length PEX38 and 0 to 5000 nM for the 65-134 amino acid region. The bead concentration and buffer were as described above. The assay was performed in three biological replicates, with 3 technical replicates each.

An AlphaScreen-based complex assay was performed to demonstrate the bridging function of PEX38, which results in the formation of a ternary complex involving PEX19, PEX38, and Hip. In this assay, 5 µL of 100 nM PEX19-His was added to a 384-well plate, followed by the addition of 5 µL of serially diluted untagged proteins, either GST or GST-PEX38. The dilution series consisted of 5 points, ranging from 0 to 400 nM for both proteins, with GST serving as a control. Following a 15-min incubation, 5 µL of 100 nM Strep-Hip was added to the pre-incubated mixture and incubated for an additional 30 min. Subsequently, 5 µL of AlphaScreen Nickel-chelate acceptor beads (cat. no. 6760619C, revvity) were added to the mixture, followed by a 15-min incubation at room temperature. Finally, 5 µL of AlphaScreen Strep-Tactin donor beads (cat. no. AS106D, revvity) were added, and the complete 25 µL reaction solutions were incubated for 45 min at room temperature in the dark. The Alpha signals were captured using a Cytation 5 plate reader with a gain value of 180. The binding assay was performed in three biological replicates, with 6 technical replicates each, to ensure the robustness and reliability of the results.

Protein sample preparation for structural characterization

PEX constructs were transformed into *E. coli* BL21 (DE3) cells and expressed in LB or isotope-enriched M9 minimal medium. Uniformly ^{15}N or ^{15}N, ^{13}C labeled proteins were expressed in H_2O M9 minimal medium supplemented with 50 µg/mL kanamycin, 1 g/L [U-^{15}N] ammonium chloride and 2 g/L hydrated [U-^{13}C] glucose as the sole sources of nitrogen and carbon, respectively. After transformation, single colonies were picked randomly and cultured in the medium of choice overnight at 37°C. The next day, cultures were diluted to an optical density of 600 nm (OD_{600}) of 0.1 and grown up to a OD_{600} of 0.4-0.6. Protein expression was induced with 0.5 mM IPTG and was carried out for 4 h at 37°C.

The cells were harvested by centrifugation at 6,000 g for 20 min at 4°C. For protein purification the cell pellets were resuspended in lysis buffer (50 mM Tris pH 7.5, 300 mM NaCl, 20 mM imidazole) substituted with lysozyme (from chicken), DNAse and protease inhibitor mix (Serva, Heidelberg, Germany) and lysed by pulsed sonication (10 min, 40% power, large probe, Fisher Scientific model 550) followed by centrifugation at 38,000 g for 45 min. All proteins were purified using gravity flow Ni-NTA (Qiagen, Monheim, Germany) affinity chromatography. The supernatant of the lysate was incubated with Ni-NTA beads (2 mL/1 L culture) for 20 min at 4°C, while rotating. Subsequently, the protein-bound beads were washed with 7 column volumes (CV) high salt buffer (50mM Tris pH 7.5, 750 mM NaCl, 20 mM imidazole) and 10 CV wash buffer (50 mM Tris pH 7.5, 300 mM NaCl, 20 mM imidazole). The elution was performed with 3-5 CV elution buffer (50 mM Tris pH 7.5, 300 mM NaCl, 500 mM imidazole). Dialysis and SUMO cleavage were executed overnight at 4°C in 20 mM Tris pH 7.5, 150 mM NaCl. Further purification was performed with a reverse Ni-NTA column where the flow through containing the cleaved protein of interest was collected and concentrated for size exclusion chromatography using a Superdex S75, 16/600 (Cytiva, Marlborough, US).

NMR spectroscopy and structure calculation

NMR data were collected using Bruker Avance III or Avance NEO spectrometers operating at 900, 950, or 1200 MHz, equipped with cryogenic probes. All NMR experiments were conducted in NMR buffer (20 mM NaP, pH 6.5, 100 mM NaCl, 1 mM DTT, and 10% D2O) at 298 K in a 5 mm diameter tube. All NMR spectra were processed using Topspin (Bruker Biospin, Rheinstetten, Germany) or NMRPipe and analyzed using CcpNMR Analysis 2.4.2 (24).

The backbone resonances of ^{15}N and ^{13}C labeled *Tb*PEX38 (65-134) and *Tb*PEX19 (1-50-SGGY) were assigned based on heteronuclear 2D and 3D experiments, including ^{1}H-^{15}N-HSQC, HNCA, HN(CO)CA, CBCA(CO)NH, HNCACB, HNCO, HN(CA)CO (25). ^{1}H and ^{13}C side chain assignments were obtained using ^{1}H-^{13}C-HSQC constant time and carbon, as well as proton-evolved HCC(CO)NH (25) and HCCH-TOCSY (hcchdigp3d, Bruker) experiments. The peptide backbone of free *Tb*PEX38 (65-134) and *Tb*PEX19 (1-50-SGGY) were assigned at concentrations of 360 µM and 380 µM, respectively. Backbone and side chain assignments of double-labeled *Tb*PEX38 (65-134) and *Tb*PEX19 (1-50-SGGY) complexed with non-labeled *Tb*PEX19 (1-50-SGGY) and *Tb*PEX38 (65-134) were obtained at concentrations of 400 µM for the double-labeled and 800-1000 µM for the non-labeled protein.

{^{1}H}-^{15}N heteronuclear NOE (hetNOE) experiments were performed using the pulse sequence hsqcnoef3gpsi (Bruker, Avance version 12.01.11) with a 4.5 s interscan delay, under the same conditions used for backbone and side chain assignments. NOE values are reported as the ratio of peak heights in experiments with and without proton saturation (hetNOE = I_{sat}/I_0).

Binding studies via NMR titration experiments were conducted at a reference protein concentration of 100 µM. Each titration point was prepared as an individual sample to avoid dilution effects. The protein ligands were added in increasing concentrations up to an 8-fold excess. The chemical shift perturbation ($\Delta\delta_{avg}$) was calculated by using formula $\Delta\delta_{avg}$=[$(\Delta\delta_H)^2$+$(\Delta\delta_N$*0.159$)^2$]

Inter- and Intradistance restraints were obtained with ^{15}N and ^{13}C edited 3D NOESY experiments (25), respectively. Automated peak picking of NOESY spectra was performed with the program Artina (26) and peak lists were manually cleaned from artefacts using the CcpNMR Analysis 2.4.2 software package (24). Resonance assignments, TALOS angle restraints (27) and cleaned NOESY peak lists from all four samples were combined and used as input for a structure calculation with Cyana (version 3.98.15; (28)) leading to an automated NOESY peak list assignment of 90+ %. A total of 2757 unambiguous NOE distance restraints (**for details, see Table S2**) were detected and used to calculate a bundle of 100 conformers, from which the 20 with the lowest Cyana target function were selected for refinement in implicit water in the program amber20 (29) and used to represent the structure ensemble.

Isothermal Titration Calorimetry (ITC)

Isothermal titration calorimetry (ITC) measurements of 40 µM *Tb*PEX38 with 440µM *Tb*PEX19 were performed as triplicates at 25°C using a MicroCal PEAQ-ITC (Malvern Instruments Ltd. U.K) calorimeter. Buffer conditions were 20 mM Tris pH 7.5, 50 mM NaCl. For all titrations, a titrant dilution control experiment was performed and subtracted before the data were fitted to a one-site binding model using the Malvern Analysis software.

Subcellular fractionation by density gradient centrifugation

Subcellular fractionation by density gradient centrifugation was employed to analyze the distribution of PEX38 and other organellar markers across the gradient's fractions in *Trypanosoma* PCF cells, as previously described in (30). The cells were ruptured with silicon carbide in a pre-cooled mortar,

followed by differential centrifugation, which yielded a cytosol-enriched fraction and an organelle-enriched pellet. Density gradient centrifugation was then applied to further investigate the distribution of PEX38 within the subcellular compartments.

Subcellular fractionation using digitonin treatment

Subcellular fractionation was performed on *Trypanosoma* PCF cells expressing GFP-tagged constructs, including GFP, GFP-PTS1, GFP-PEX38, and PEX38-GFP, after induction with tetracycline for approximately ~24 h. The cells expressing the proteins of interest were treated with digitonin at a final concentration of 0.1 mg/mg of protein for 3-5 min at 37°C, selectively permeabilizing the plasma membrane. This was followed by centrifugation at 20,800 g (Rotor F45-24-11, Eppendorf) for 15 min at 4°C, which separated the samples into a cytosol-enriched supernatant and an organellar pellet. The samples collected from each step were then analyzed using immunoblotting.

RNA interference and digitonin fractionation

The double-stranded stem-loop PEX38 RNAi construct was cloned into a *Trypanosoma* expression plasmid p2T7-177, as described in the '**cloning**' section. This construct was then genomically integrated into the BSF or PCF *Trypanosoma* cells via transfection, as outlined in the '***Trypanosoma* cell culture and transfection**' section. Positive clones were subjected to a survival analysis. RNAi was induced by the addition of tetracycline to the transfected *Trypanosoma* cells, which were cultured at a density of 2×10^5 cells/mL for BSF and 1×10^6 cells/mL for PCF. DMSO-treated cells served as negative controls. After 24 h of incubation, the cell counts were recorded, and the cells were diluted back to the initial densities with the addition of tetracycline. Cultures with densities below the initial values were incubated further without dilution or additional tetracycline. The growth of the transfected cell lines treated with DMSO, or tetracycline, was monitored for 4 days in the case of BSF and 9 days for PCF. The experiment was performed in three biological replicates, with two technical replicates for BSF and PCF. The cumulative growth curves were plotted on a logarithmic scale using GraphPad Prism 10.

Biochemical fractionation using digitonin was performed on day 2 RNAi-harvested samples, including both DMSO-treated and RNAi-induced cells, as described previously (30). The resulting supernatant was analyzed by immunoblotting using various antibodies, including markers such as enolase, GAPDH, phosphofructokinase, glycerol-3-phosphate dehydrogenases (GPD), aldolase, hexokinase, and MtHsp70.

Complementation assay

Functional complementation analysis was performed as described in detail in (14). For the *in-cellulo* complementation assay, a codon-exchanged, RNAi-resistant PEX38 gene with an N-terminal 2×FLAG tag was custom-synthesized and obtained from GeneCust, France. Using this as a template, mutants were generated with the primers and strategies described in **Tables S2** and **S3**. These tetracycline-inducible, codon-exchanged constructs, comprising wild-type or mutant 2×FLAG-PEX38, were then transfected into the previously generated BSF PEX38 RNAi cells, as described in the ***Trypanosoma* cell culture and transfection** section. In this system, tetracycline induction results in the depletion of endogenous PEX38 and the simultaneous overexpression of the ectopic, codon-exchanged 2×FLAG-PEX38 protein, either wild-type or mutant. Two controls were used to validate the complementation assay: a negative control transfected with an empty vector and a positive control transfected with the wild-type 2×FLAG-PEX38 construct. Upon tetracycline induction, the negative control cannot restore the growth defect caused by PEX38 RNAi, while the positive control should result in near-normal growth, as PEX38 RNAi is functionally

complemented by the codon-exchanged, ectopically expressed wild-type PEX38. Growth analysis was performed as described in the **RNA interference and digitonin fractionation** section, with cells transfected with wild-type and mutant constructs monitored for 3 days. Based on the growth behavior, the essentiality of various PEX38 mutations can be assessed.

Microscopy

Trypanosoma cell lines harboring various tetracycline-inducible GFP-tagged constructs were generated, including GFP, GSP-SKL, GFP-PEX38, and PEX38-GFP. These cell lines were either induced with 1 µg/mL tetracycline or treated with DMSO as a negative control. The cells were then sedimented, fixed in 4% paraformaldehyde in PBS at 4°C for 15 min, and processed for imaging as previously described in (4, 30). The fixed *Trypanosoma* cells were stained with an antibody against the glycosomal marker aldolase (1:500), and the stained cells were visualized and imaged using a Carl Zeiss microscope equipped with Zen 3.6 software. The acquired images were processed using deconvolution and merged. Subsequently, the processed images were analyzed with Zeiss Zen 3.2 software (blue edition).

Synthesis of cDNA

From the BSF day 1 and day 2 RNAi cells, either induced with 1 µg/mL tetracycline or treated with DMSO as a negative control, cells were harvested and RNA isolated using the NucleoSpin RNA kit (cat no. 740955.50, Macherey-Nagel) following the protocol for RNA purification from cultured cells and tissues. cDNA was prepared using the RevertAid First Strand cDNA Synthesis Kit (cat no. K1621, Thermo Scientific) with 400 ng of total RNA. The semi-quantitative analysis of tubulin (RE7000, RE7001) and PEX38 (RE7528, RE7723) mRNA levels were carried out by routine PCR with the prepared cDNA. The primer sequences used for this analysis are described in **Table S5**.

Bioinformatics analysis

The protein sequences for Human SGTA, SGTB, ECD/Human suppressor of GCR two, and Yeast (*S. cerevisiae*) Sgt2 were obtained from the UniProt database, with the respective IDs O43765, Q96EQ0, O95905, and Q12118. Additionally, the protein sequence for the Human Hsc70-interacting protein (Hip) was also retrieved from the UniProt database with ID P50502. The protein sequences of the identified PEX38 orthologs from the Trypanosomatid parasites *T. brucei*, *T. cruzi*, *L. donovani*, *L. mexicana*, *L. major*, and *L. infantum* were obtained from the TriTrypDB database with accession IDs Tb927.6.4000/ Tb427_060045000, TcCLB.511737.10, and LdBPK_302740.1, LmxM.29.2740, LmjF.30.2740, and LINF_300032600 respectively. For *Diplonema papillatum*, the sequence was retrieved from GenBank using the corresponding accession number KAJ9472742.1, identified through a BLAST search. The protein sequence of the *T. brucei* Hip was also retrieved from the TriTrypDB database with ID Tb427_030056900. The domain architecture and family of the proteins were predicted using the InterPro Scan tool (31). The multiple sequence alignment was performed with the Clustal Omega tool (32) and visualised using Jalview software (v 2.11.0) with a percentage identity colour scheme and a conservation threshold of 30%. A percentage identity and similarity matrix of the proteins was also calculated using the SIAS tool with the BLOSUM62 matrix (http://imed.med.ucm.es/Tools/sias.html).

GET/TRC pathway orthologs were identified using BLAST, InterPro, PantherDB domain searches, and OrthoCML, while structural similarity searches were performed with Foldseek. The protein sequences for Get1 and Get3 were obtained from UniProt or TriTrypDB. Get1 orthologs include *Hs*Get1 (O00258), *Sc*Get1 (P53192), and *Bodo saltans* (BSAL_19620). Get3 orthologs include *Hs*Get3 (O43681), *Sc*Get3 (Q12154), *T.cruzi* (*Tc*Get3, TcCLB.510101.490), *L. donovani* (*Ld*Get3, LdBPK_110710.1.1) and *B. saltans* (*Bs*Get3, BSAL_77145).

Immunoblotting

The samples or cell lysate to be analyzed were denatured in Laemmli buffer for 5 min at 95 °C and separated by SDS-PAGE. Following gel electrophoresis, immunoblotting was performed as described in (30) . The primary antibodies used in this study were mouse anti-GFP, FLAG, MtHSP70 and human alpha-tubulin (which shows cross-reactivity with *Trypanosoma* tubulin) and rabbit anti-*Trypanosoma* Aldolase, Enolase, Hexokinase, GAPDH, GIM5, PEX11, PFK, G3PDH/GPD, VDAC and PEX38. The dilutions, buffers, and secondary antibodies are described in (30), except for the FLAG and PEX38 antibodies. The PEX38 antibody was generated in this study using a label-free PEX38 1-134 antigen and was subsequently affinity-purified by Eurogentec. The FLAG (1:2,000, Sigma-Aldrich) and PEX38 antibodies (1 µg/mL) were prepared in PBS-T (Tween-20 0.05%) buffer with 3% BSA. The immunoblots were scanned using the LI-COR Odyssey Infrared Imaging System and analyzed with Image Studio version 5.2.

Statistical analysis

Statistical analysis for AlphaScreen results was performed using 2-way ANOVA with Šídák's multiple comparisons test within each row, comparing columns with respective controls. The analysis was based on the values obtained from three independent biological replicates, each with six technical replicates. For the competition assay, the values were obtained from three independent biological replicates, each with three technical replicates. These values were initially transformed to log values using the function X=Log (X), which were further analyzed by non-linear regression, i.e., curve fitting with the equation for log(inhibitor) vs. response -- Variable slope (four parameters) to determine the IC 50. All the analysis was performed using GraphPad Prism (Version 10.3.0).

SI References

1. F. Hong *et al.*, RankProd: a bioconductor package for detecting differentially expressed genes in meta-analysis. *Bioinformatics* **22**, 2825-2827 (2006).
2. R. A. Laskowski, J. A. Rullmannn, M. W. MacArthur, R. Kaptein, J. M. Thornton, AQUA and PROCHECK-NMR: programs for checking the quality of protein structures solved by NMR. *J Biomol NMR* **8**, 477-486 (1996).
3. V. C. Kalel *et al.*, Evolutionary divergent PEX3 is essential for glycosome biogenesis and survival of trypanosomatid parasites. *Biochimica et biophysica acta. Molecular cell research* **1866**, 118520 (2019).
4. C. K. Krishna *et al.*, Molecular basis of the glycosomal targeting of PEX11 and its mislocalization to mitochondrion in trypanosomes. *Frontiers in cell and developmental biology* **11**, 1213761 (2023).
5. D. Kerssen *et al.*, Membrane association of the cycling peroxisome import receptor Pex5p. *J Biol Chem* **281**, 27003-27015 (2006).
6. D. Effelsberg, L. D. Cruz-Zaragoza, W. Schliebs, R. Erdmann, Pex9p is a new yeast peroxisomal import receptor for PTS1-containing proteins. *Journal of cell science* **129**, 4057-4066 (2016).
7. A. Halbach *et al.*, Function of the PEX19-binding site of human adrenoleukodystrophy protein as targeting motif in man and yeast. PMP targeting is evolutionarily conserved. *J Biol Chem* **280**, 21176-21182 (2005).
8. M. Dawidowski *et al.*, Inhibitors of PEX14 disrupt protein import into glycosomes and kill Trypanosoma parasites. *Science* **355**, 1416-1420 (2017).
9. C. Li *et al.*, FastCloning: a highly simplified, purification-free, sequence- and ligation-independent PCR cloning method. *BMC biotechnology* **11**, 92 (2011).
10. S. Redmond, J. Vadivelu, M. C. Field, RNAit: an automated web-based tool for the selection of RNAi targets in Trypanosoma brucei. *Molecular and biochemical parasitology* **128**, 115-118 (2003).
11. R. Brun, Schonenberger, Cultivation and in vitro cloning or procyclic culture forms of Trypanosoma brucei in a semi-defined medium. Short communication. *Acta tropica* **36**, 289-292 (1979).
12. H. Hirumi, K. Hirumi, Continuous cultivation of Trypanosoma brucei blood stream forms in a medium containing a low concentration of serum protein without feeder cell layers. *The Journal of parasitology* **75**, 985-989 (1989).
13. C. K. Krishna, R. Erdmann, V. C. Kalel, "Mapping Trypanosoma Protein Interactome by Proximity-Dependent Biotinylation" in Euglenozoa: Methods and Protocols, Volume 1*,* P. A. M. Michels, M. L. Ginger, A. Karnkowska, L.-I. McCall, A. M. Silber, Eds. (Springer US, New York, NY, 2026), 10.1007/978-1-0716-5142-1_18, pp. 343-359.
14. C. K. Krishna, R. Erdmann, V. C. Kalel, "Tetracycline-Inducible Functional Complementation Analysis in Trypanosoma Parasites" in Euglenozoa: Methods and Protocols, Volume 2*,* P. A. M. Michels, M. L. Ginger, A. Karnkowska, L.-I. McCall, A. M. Silber, Eds. (Springer US, New York, NY, 2026), 10.1007/978-1-0716-5146-9_7, pp. 95-111.
15. I. Gromova, J. Celis, "Protein Detection in Gels by Silver StainingA Procedure Compatible with Mass Spectrometry" in Cell Biology*,* J. E. Celis, Ed. (Academic Press, Burlington, 2006), 10.1016/b978-012164730-8/50212-4, pp. 219-223.
16. M. Korner *et al.*, p97/VCP is required for piecemeal autophagy of aggresomes. *Nat Commun* **16**, 4243 (2025).

17. J. Cox, M. Mann, MaxQuant enables high peptide identification rates, individualized p.p.b.-range mass accuracies and proteome-wide protein quantification. *Nat Biotechnol* **26**, 1367-1372 (2008).

18. J. Cox *et al.*, Andromeda: a peptide search engine integrated into the MaxQuant environment. *Journal of proteome research* **10**, 1794-1805 (2011).

19. J. Bender, W. W. D. Mühlhäuser, J. P. Zimmerman, F. Drepper, B. Warscheid, Autoprot: Processing, Analysis and Visualization of Proteomics Data in Python. *bioRxiv* 10.1101/2024.01.18.571429, 2024.2001.2018.571429 (2024).

20. W. S. Cleveland, S. J. Devlin, Locally Weighted Regression - an Approach to Regression-Analysis by Local Fitting. *Journal of the American Statistical Association* **83**, 596-610 (1988).

21. F. Del Carratore *et al.*, RankProd 2.0: a refactored bioconductor package for detecting differentially expressed features in molecular profiling datasets. *Bioinformatics (Oxford, England)* **33**, 2774-2775 (2017).

22. E. Yifrach *et al.*, Systematic multi-level analysis of an organelle proteome reveals new peroxisomal functions. *Mol Syst Biol* **18**, e11186 (2022).

23. A. Neuhaus *et al.*, A novel Pex14 protein-interacting site of human Pex5 is critical for matrix protein import into peroxisomes. *J Biol Chem* **289**, 437-448 (2014).

24. W. F. Vranken *et al.*, The CCPN data model for NMR spectroscopy: development of a software pipeline. *Proteins* **59**, 687-696 (2005).

25. M. Sattler, J. Schleucher, C. Griesinger, Heteronuclear multidimensional NMR experiments for the structure determination of proteins in solution employing pulsed field gradients. *Prog Nucl Mag Res Sp* **34**, 93-158 (1999).

26. P. Klukowski, R. Riek, P. Guntert, Rapid protein assignments and structures from raw NMR spectra with the deep learning technique ARTINA. *Nat Commun* **13**, 6151 (2022).

27. Y. Shen, A. Bax, Protein backbone and sidechain torsion angles predicted from NMR chemical shifts using artificial neural networks. *J Biomol NMR* **56**, 227-241 (2013).

28. L. Buchner, P. Guntert, Increased reliability of nuclear magnetic resonance protein structures by consensus structure bundles. *Structure* **23**, 425-434 (2015).

29. D. A. Case *et al.*, The Amber biomolecular simulation programs. *Journal of computational chemistry* **26**, 1668-1688 (2005).

30. C. K. Krishna *et al.*, High-confidence glycosomal membrane protein inventory unveils trypanosomal peroxin PEX15. *Cell Rep* **44**, 115614 (2025).

31. T. Paysan-Lafosse *et al.*, InterPro in 2022. *Nucleic Acids Res* **51**, D418-D427 (2023).

32. F. Madeira *et al.*, Search and sequence analysis tools services from EMBL-EBI in 2022. *Nucleic Acids Res* **50**, W276-W279 (2022).
